# The Coexistence of Bacterial Species Restructures Biofilm Architecture and Increases Tolerance to Antimicrobial Agents

**DOI:** 10.1128/spectrum.03581-22

**Published:** 2023-02-27

**Authors:** Jiajun Dong, Luhan Liu, Liying Chen, Yuqiang Xiang, Yabin Wang, Youbao Zhao

**Affiliations:** a College of Veterinary Medicine, Henan Agricultural University, Zhengzhou, Henan, China; b Key Laboratory for Animal-derived Food Safety of Henan Province, Zhengzhou, Henan, China; University of Reims Champagne-Ardenne, Biomatériaux et Inflammation en site Osseux

**Keywords:** *Enterococcus faecalis*, polymicrobial biofilm, extracellular matrix, biofilm architecture, nutrient stress, interspecies interactions, biofilms, environmental microbiology

## Abstract

Chronic infections caused by polymicrobial biofilms are often difficult to treat effectively, partially due to the elevated tolerance of polymicrobial biofilms to antimicrobial treatments. It is known that interspecific interactions influence polymicrobial biofilm formation. However, the underlying role of the coexistence of bacterial species in polymicrobial biofilm formation is not fully understood. Here, we investigated the effect of the coexistence of Enterococcus faecalis, Escherichia coli O157:H7, and Salmonella enteritidis on triple-species biofilm formation. Our results demonstrated that the coexistence of these three species enhanced the biofilm biomass and led to restructuring of the biofilm into a tower-like architecture. Furthermore, the proportions of polysaccharides, proteins, and eDNAs in the extracellular matrix (ECM) composition of the triple-species biofilm were significantly changed compared to those in the E. faecalis mono-species biofilm. Finally, we analyzed the transcriptomic profile of E. faecalis in response to coexistence with E. coli and S. enteritidis in the triple-species biofilm. The results suggested that E. faecalis established dominance and restructured the triple-species biofilm by enhancing nutrient transport and biosynthesis of amino acids, upregulating central carbon metabolism, manipulating the microenvironment through “biological weapons,” and activating versatile stress response regulators. Together, the results of this pilot study reveal the nature of E. faecalis-harboring triple-species biofilms with a static biofilm model and provide novel insights for further understanding interspecies interactions and the clinical treatment of polymicrobial biofilms.

**IMPORTANCE** Bacterial biofilms possess distinct community properties that affect various aspects of our daily lives. In particular, biofilms exhibit increased tolerance to chemical disinfectants, antimicrobial agents, and host immune responses. Multispecies biofilms are undoubtedly the dominant form of biofilms in nature. Thus, there is a pressing need for more research directed at delineating the nature of multispecies biofilms and the effects of the properties on the development and survival of the biofilm community. Here, we address the effects of the coexistence of Enterococcus faecalis, Escherichia coli, and Salmonella enteritidis on triple-species biofilm formation with a static model. In combination with transcriptomic analyses, this pilot study explores the potential underlying mechanisms that lead to the dominance of E. faecalis in triple-species biofilms. Our findings provide novel insights into the nature of triple-species biofilms and indicate that the composition of multispecies biofilms should be a key consideration when determining antimicrobial treatments.

## INTRODUCTION

Bacterial biofilms are clinically important due to their association with chronic and device-related persistent infections, such as infections in patients with cystic fibrosis, urinary tract infections, and endocarditis (1). Approximately 80% of microbial infections are caused by bacteria in biofilms, and biofilms in nature usually contain multiple species (2, 3). More importantly, bacteria in multispecies biofilms frequently exhibit enhanced resistance and tolerance to antibiotics, as well as resistance to the host immune response and infection persistence (4–6). However, the mechanisms underlying the formation of multispecies biofilms are complex.

The factors affecting multispecies biofilm succession are currently believed to include environmental variability, social interactions (including but not limited to the classic “rock-paper-scissors” strategy for spatial colonization, cross-feeding, and metabolic trade-off), and local spatial organization (7, 8). Of these, the latter two are critical to the stability of multispecies communities and are involved in maintaining the diversity of competing ecosystems (8). In addition, multispecies biofilms typically exhibit various spatial structures, such as mixed cell lineages, segregated microcolonies, and layered structures (9, 10). These specific spatial structures shape the functions and community composition within multispecies biofilms (11). Therefore, understanding the social interactions and the spatial structure of multispecies communities is important for elucidating the formation and development of multispecies biofilms.

Enterococcus faecalis is frequently isolated from a variety of microbial infection sites, including the urinary tract, burns, and diabetic foot ulcers (12), and it commonly exhibits the ability to form biofilms (13). For instance, E. faecalis is frequently found in polymicrobial communities associated with urinary tract infections and catheter-associated urinary tract infections (CAUTI). In the course of CAUTI, E. faecalis and uropathogenic Escherichia coli are frequently isolated together (14). Additionally, E. faecalis typically enhances the formation of multispecies biofilms. For example, AI-2 produced by E. faecalis attracts E. coli to the aggregate, and L-ornithine secreted by E. faecalis significantly enhances E. coli biofilm growth and survival both in *vivo* and in *vitro* (15–17). However, the dynamics, stability, and mechanisms underlying the interactions between E. faecalis and other bacteria in biofilms are poorly understood.

As foodborne pathogens, E. coli O157:H7 and Salmonella enteritidis share the same niches and scenarios with E. faecalis, including those associated with nosocomial (18, 19), foodborne (20–22), and waterborne (23) infections. They are also commonly found on other abiotic surfaces, such as those in food processing settings (24–26), in addition to coexisting in the intestine. Furthermore, it has been reported that the coexistence of certain bacteria can enhance biofilm formation and alter biofilm properties, leading to effects such as enhanced tolerance to antibiotics (6), persistence of infection (27), and, consequently, increased food hygiene and safety risks (27). In this pilot study, we investigated the effects of the coexistence of E. coli, E. faecalis, and S. enteritidis on triple-species biofilm formation using a static biofilm model. We found that E. faecalis was a dominant member and played a key role in stabilizing the triple-species biofilm by restructuring it to form a tower-like structure. Transcriptome analysis revealed that the dominance of E. faecalis was a result of enhanced nutrient transport and amino acid biosynthesis, timely regulation of central carbon metabolism, utilization of “biological weapons,” and activation of versatile stress response regulators. Our study provides insights for further investigating the mechanism of interspecies interactions in multispecies biofilms and the clinical treatment of multispecies biofilm-associated infections.

## RESULTS

### Coexistence of E. faecalis, E. coli, and S. enteritidis enhances biofilm biomass.

**FIG 1:**
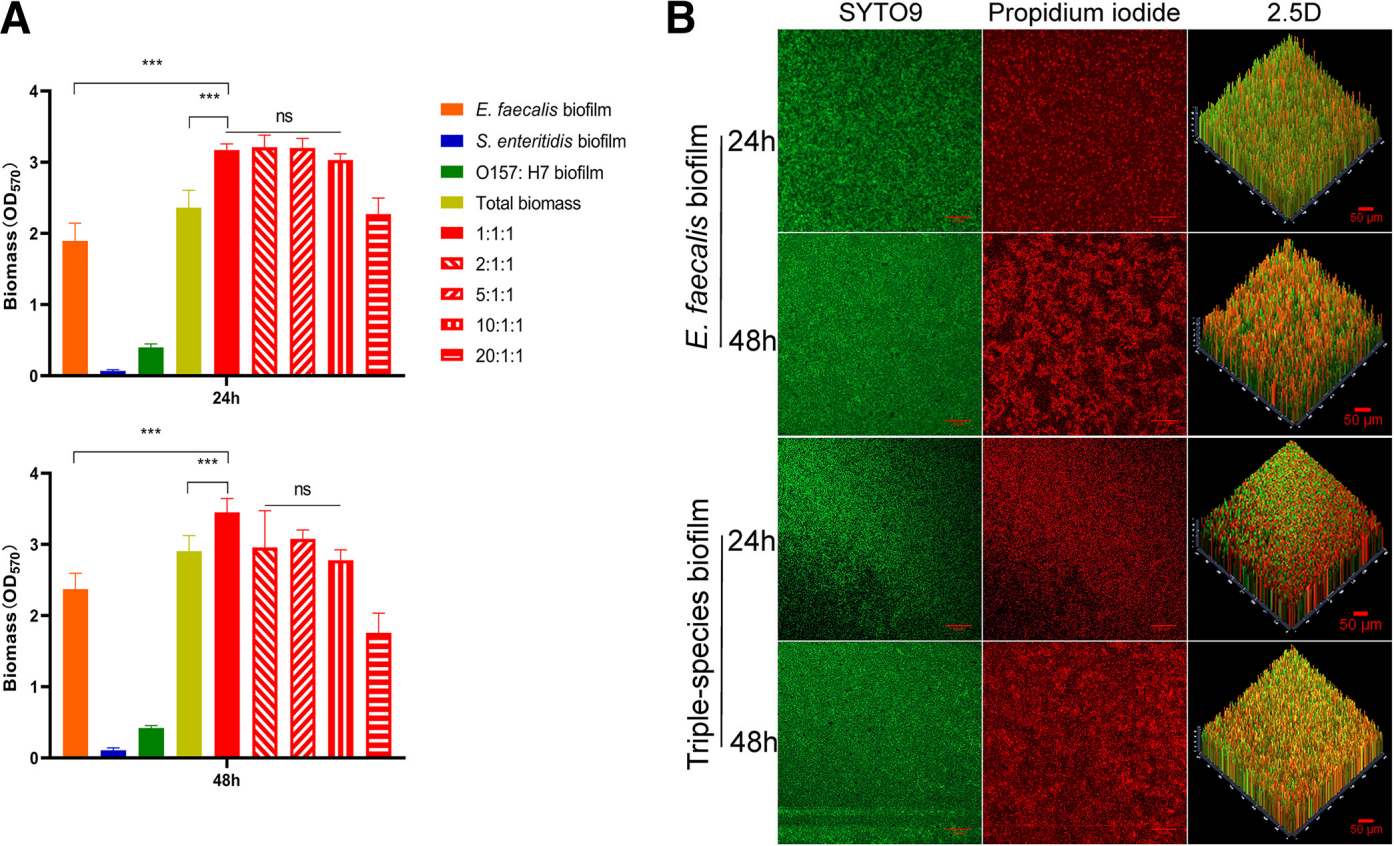
Coexistence of E. faecalis, E. coli O157:H7, and S. enteritidis enhances the biofilm formation. (A) E. faecalis, E. coli O157:H7, and S. enteritidis mixed at the indicated ratios or by themselves were cultured for 24 h and 48 h, and the biomass of biofilms was determined by OD_570_. Total biomass is the total biomass of three mono-species biofilms. (B) Visualization of live and dead cells in biofilms by CLSM. The formed biofilms were stained using the LIVE/DEAD BacLight Bacterial Viability kit. SYTO9 green for DNA of living cells, and propidium iodide red for DNA in dead cells. Images are representative of at least three independent experiments. Bars, 50 μm.

E. faecalis, E. coli, *and* S. enteritidis are foodborne pathogens that can together form biofilms with increased antimicrobial tolerance and infection persistence (4–6, 27). To dissect the nature of the triple-species biofilm of E. faecalis, E. coli, and S. enteritidis, we first determined the effect of the coexistence of these bacteria at various ratios (1:1:1/2:1:1/5:1:1/10:1:1, E. faecalis: E. coli O157:H7:S. enteritidis) on biofilm biomass. The results showed that the coexistence of these bacteria at different ratios enhanced the biomass of the triple-species biofilm compared to the total biomass of the three mono-species biofilms at 24 h (Fig. 1A). Strikingly, the triple-species biofilm biomass was also increased by relatively low proportions of E. coli O157:H7 and S. enteritidis (20:1:1) at 24 h. In addition, the biomass of the triple-species biofilm with the three species presented at a ratio of 1:1:1 was still significantly higher than the total biomass of the three mono-species biofilms at 48 h. However, the biomass of the triple-species biofilm with an initial ratio of 2:1:1, 5:1:1, or 10:1:1 was not significantly different from the total biomass of the three mono-species biofilms, while the triple-species biofilm with an initial ratio of 20:1:1 showed reduced biomass (Fig. 1A). The results strongly suggest that the coexistence of E. faecalis, E. coli, *and* S. enteritidis enhanced triple-species biofilm formation and that the initial ratio affected triple-species biofilm formation. Accordingly, we chose 1:1:1 as the ratio for the following assays to further investigate the triple-species biofilm.

E. faecalis biofilm formation occurs in two phases, namely, the initial phase (24 h) and mature phase (48 h). To confirm the effect of the coexistence of these bacteria on biofilm formation, we further measured biofilm formation with 0.1% crystal violet staining and confocal laser scanning microscopy (CLSM) imaging separately. The crystal violet staining results showed that the triple-species biofilm biomass increased approximately 1.7- and 1.45-fold compared to the biomass of the E. faecalis mono-species biofilm at 24 h and 48 h, respectively (Fig. 1A). CLSM imaging clearly showed that the triple-species biofilm was denser than the E. faecalis mono-species biofilm in both phases (Fig. 1B). In addition, based on the live/dead cell staining results from CLSM imaging, the triple-species biofilm at 48 h contained more live and dead cells compared to the other biofilms (Fig. 1B). Again, these results confirmed that the coexistence of these bacteria enhanced triple-species biofilm formation.

Interestingly, coculturing E. faecalis with either E. coli O157:H7 or S. enteritidis also significantly enhanced double-species biofilm formation (Fig. S1). However, the biomass of the double-species biofilm formed by E. coli O157:H7 and S. enteritidis was lower (Fig. S1). These pilot results indicated that E. faecalis plays a key role in interspecies interactions and multispecies biofilm formation.

### The triple-species biofilm forms a distinct tower-like architecture.

**FIG 2:**
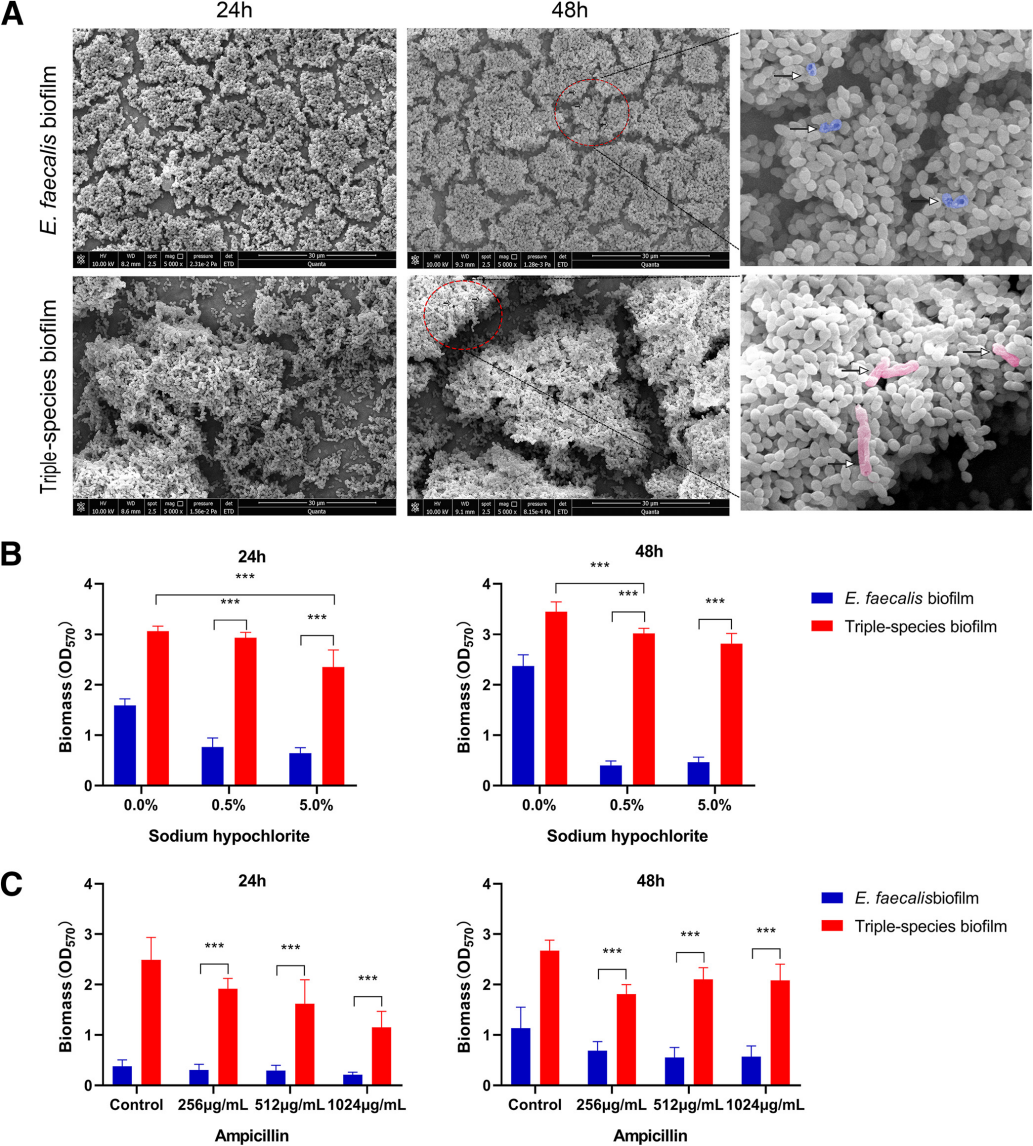
Triple-species biofilms form a tower-like architecture with increased tolerance to antimicrobial agents. (A) SEM imaging of architectures of the single E. faecalis biofilms and the triple-species biofilms. Red dashed circles represent lysed bacterial cells. The rightmost images represent a magnified view of the red dashed circle in 48 h biofilms. Blue represents lysed E. faecalis cell; pink represents lysed rod-shaped cell. The images are representative of at least three independent experiments. (B–C) Triple-species biofilms show increased tolerance to sodium hypochlorite (B) and ampicillin (C). Biofilms after 24h or 48h culturing were treated with a range of concentrations of sodium hypochlorite for 5 min and ampicillin for 24 h. Biofilm biomass was determined by OD_570_.

To further dissect the architecture of the triple-species biofilm, we imaged it using scanning electron microscopy (SEM). Surprisingly, the triple-species biofilm formed a complex tower-like structure with tight cell–cell contact, while the E. faecalis mono-species biofilm formed a flat structure (Fig. 2A). The tower-like structure was dominated by E. faecalis, while E. coli O157:H7 and S. enteritidis were observed in very small amounts (Fig. 2A). These results suggested that E. faecalis-harboring triple-species biofilms form a tower-like architecture.

Biofilm formation is known to be closely associated with tolerance to antimicrobials in chronic and device-related infections. To examine the tolerance of E. faecalis-harboring triple-species biofilms to antimicrobial agents, we treated the triple-species biofilm cultured for 24 h or 48 h with various concentrations of the commonly used disinfectant sodium hypochlorite and antibiotic ampicillin. The results showed that the triple-species biofilm exhibited much greater tolerance to both sodium hypochlorite and ampicillin than the E. faecalis mono-species biofilm (Fig. 2B and C). Together, these observations revealed that the coexistence of E. faecalis, E. coli O157:H7 and S. enteritidis results in the formation of triple-species biofilms with a tower-like architecture and enhanced tolerance to antimicrobial agents.

### E. faecalis dominates triple-species biofilms.

**FIG 3:**
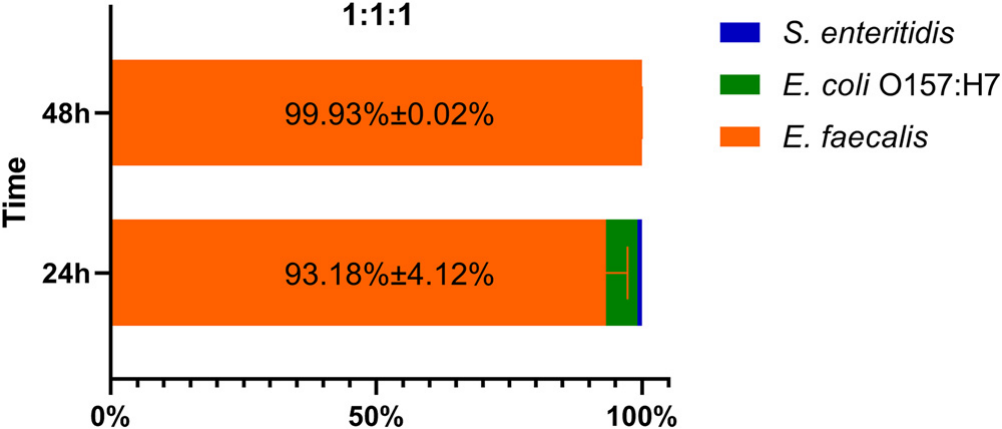
Proportion of E. faecalis to the total number of bacteria in the triple-species biofilms at 24 h and 48 h. The initial ratio of E. faecalis, E. coli O157:H7, and S. enteritidis is 1:1:1.

Bacterial cells generally account for 10% of the biofilm biomass. Our CLSM imaging results showed that the triple-species biofilm had a higher density of both live and dead cells than the E. faecalis mono-species biofilm (Fig. 1C). Furthermore, the SEM results indicated that spherical E. faecalis cells dominated the triple-species biofilm (Fig. 2A). To further determine the proportion of live bacterial cells in the community in the triple-species biofilm, we enumerated the colony-forming unit (CFU) of E. faecalis, E. coli O157:H7, and S. enteritidis on corresponding selection media on which only one of these bacterial species could grow. In the triple-species biofilm with a starting coculture ratio of 1:1:1 for E. faecalis, E. coli O157:H7, and S. enteritidis, E. faecalis dominated the community, with a proportion of 93.18% at 24 h and 99.18% at 48 h (Fig. 3). To exclude the potential effect of the starting coculture ratio on the variation in live cell proportion, we verified the dominance of E. faecalis at 24 h and 48 h in the triple-species biofilm with a starting coculture ratio of 0.5:1:1 for E. faecalis, E. coli O157:H7, and S. enteritidis. The results consistently demonstrated that E. faecalis dominated the triple-species biofilm independent of the starting coculture ratio (Fig. S2).

### ECM components of the triple-species biofilm show phase-dependent variation.

**FIG 4:**
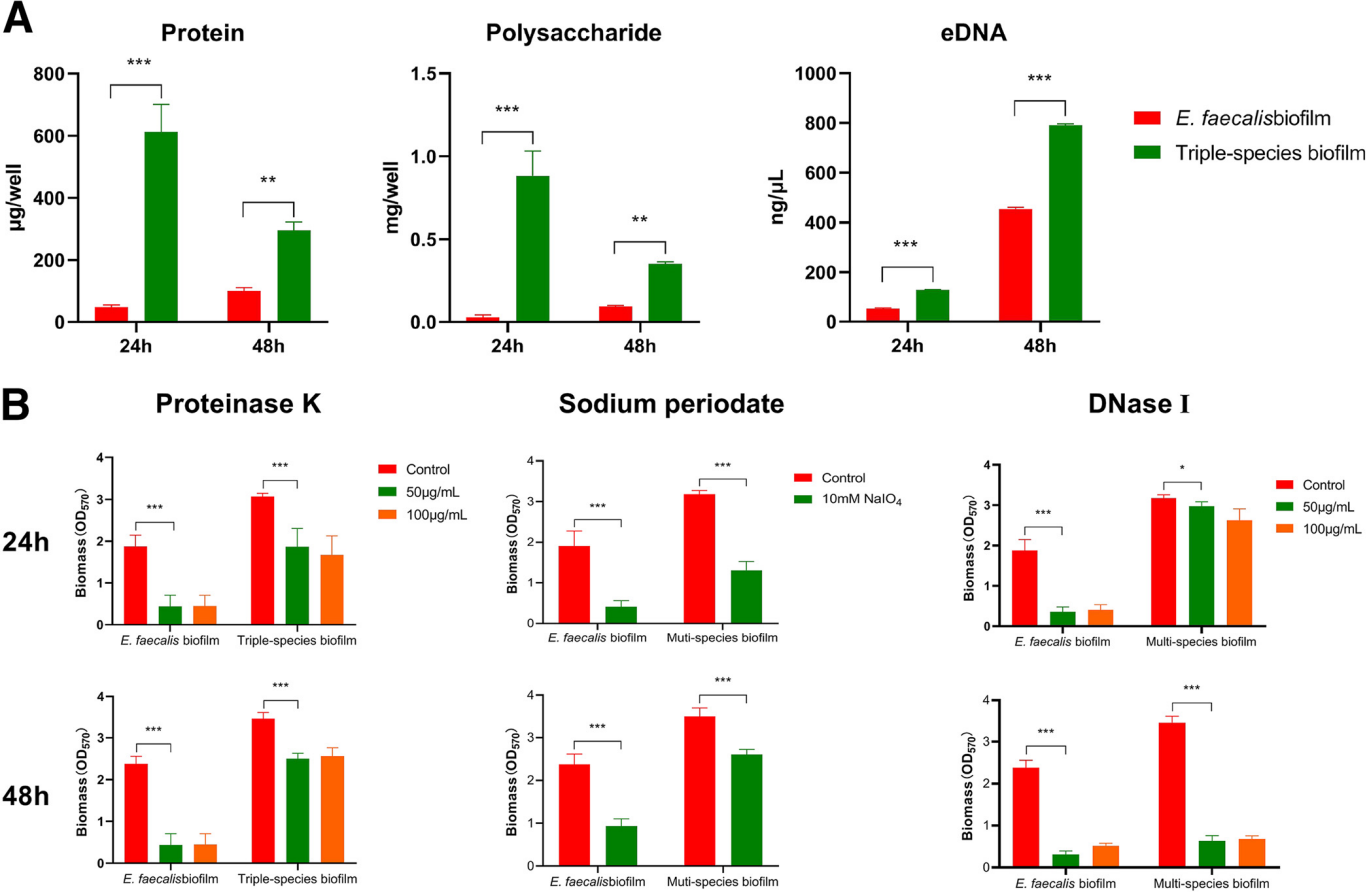
The changes in major components of ECM are related to triple-species biofilm formation. (A) Changes in the major components of ECM, including proteins, polysaccharides, and eDNA in biofilms at 24 h and 48 h. (B) Biofilms at 24 h and 48 h were treated with proteinase K, sodium periodate, and DNase I for 24 h, respectively. Biofilm biomass was determined by OD_570_.

The extracellular matrix (ECM) consists primarily of polysaccharides, proteins, and nucleic acids, which account for approximately 90% of the biofilm biomass. SEM imaging of the triple-species biofilm showed the restructuring of the biofilm and tightened contact among cells (Fig. 2A). These observations indicated that the ECM components of the triple-species biofilm may have changed compared to those of the E. faecalis mono-species biofilm. To address this question, we analyzed the amounts of polysaccharides, proteins, and nucleic acids in triple-species biofilm. Our results showed that all three kinds of ECM components were significantly enriched in the triple-species biofilm compared to the E. faecalis mono-species biofilm at 24 h and 48 h, respectively (Fig. 4A). In the E. faecalis mono-species biofilm, all three kinds of ECM components were enriched from the initial phase (24 h) to the mature phase (48 h) of biofilm formation, indicating the accumulation of ECM components (Fig. 4A). Interestingly, the protein and polysaccharide levels were significantly decreased in the 48 h triple-species biofilm compared to the 24 h triple-species biofilm, while the extracellular DNA (eDNA) level was dramatically increased (Fig. 4A). These observations were confirmed by staining of the three ECM components (Fig. S3).

To further investigate the effects of changes in ECM components on biofilm formation, we treated the triple-species biofilm with proteinase K (proteins), sodium periodate (polysaccharides), and DNase I (eDNA). The results showed that all three treatments significantly reduced the triple-species biofilm biomass (Fig. 4B). Interestingly, treatment with proteinase K or sodium periodate had more dramatic effects on the biomass of the 24 h triple-species biofilm than on that of the 48 h biofilm, while DNase I treatment had a more dramatic effect on the 48 h triple-species biofilm than on the 24 h biofilm (Fig. 4B). Taken together, these results demonstrated that the ECM components changed significantly in the triple-species biofilm and that the changes may have contributed to biomass accumulation in the triple-species biofilm.

### Transcriptomic profiling reveals genes involved in the dominance of E. faecalis in triple-species biofilms.

**FIG 5:**
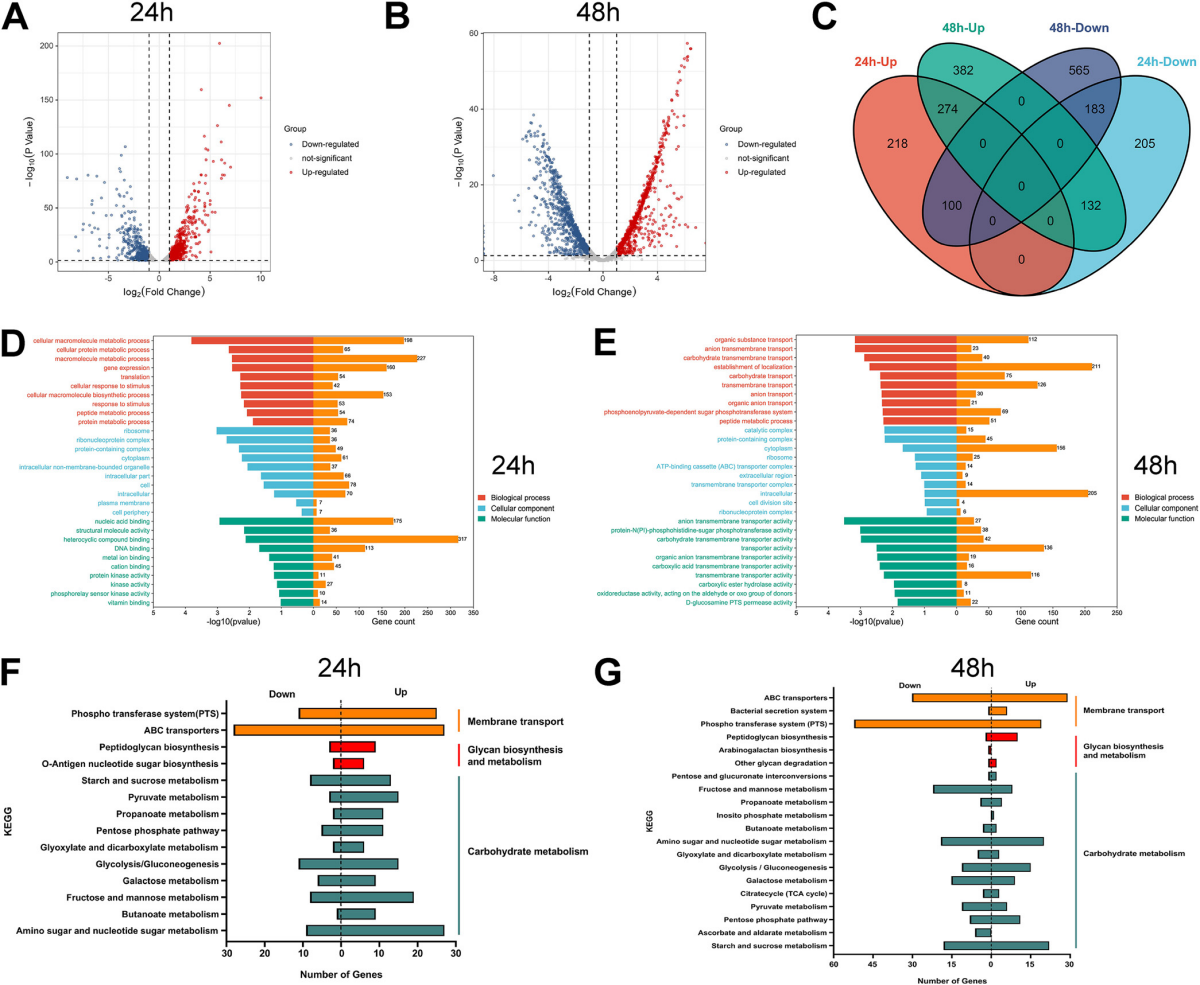
Transcriptomic analyses of E. faecalis genes in triple-species biofilms compared to that in the single E. faecalis biofilms. (A–B) Volcano plots of DEGs (triple-species biofilms versus E. faecalis biofilms) at 24 h (A) and 48 h (B). A |fold change| >2 was selected as the cutoff value for DEGs. Red for upregulated genes; blue for downregulated genes. (C) Venn diagram of DEGs at 24 h and 48 h. Up represents upregulated genes; Down represents downregulated genes. (D–E) GO enrichment analyses of DEGs at 24 h (D) and 48 h (E). (F–G) KEGG pathway enrichment analyses of DEGs at 24 h (F) and 48 h (G). Up represents upregulation; Down represents downregulation.

Given the dominance of E. faecalis in the triple-species biofilm, we decided to systematically analyze the transcriptomic changes of E. faecalis in response to coexistence with E. coli and S. enteritidis by RNA sequencing (RNA-seq). We used a static biofilm to mimic a nutritionally unsustainable environment and performed RNA-seq on E. faecalis. The RNA-seq results showed that there were 592 upregulated genes and 520 downregulated genes in the 24 h triple-species biofilm and 788 upregulated genes and 848 downregulated genes in the 48 h triple-species biofilm (Fig. 5A and B; Table S1). We next conducted qPCR analyses on the transcript levels of differentially expressed genes (DEGs), which are known to be related to biofilm formation and E. faecalis sugar metabolism (28, 29), and obtained consistent differential expression patterns with the RNA-seq data (Fig. S4). Of these DEGs from RNA-seq analyses, 274 were upregulated at both 24 h and 48 h, with the major functions involving various response regulator transcription factors, bacterial cell division, glycosyl hydrolase, PTS transport, ABC transport, WXL domain-containing protein, type II toxin-antitoxin system, and unknown functional proteins (Fig. 5C; Table S2). Notably, at 48 h in the triple-species biofilm, approximately 60.3% of the upregulated genes and 61.9% of the downregulated genes showed a more than 4-fold change in expression (Table S1).

The Gene Ontology (GO) functional enrichment analysis showed that the functions of the E. faecalis DEGs in the 24 h triple-species biofilm were mainly annotated to cellular macromolecule metabolic process, ribosome, nucleic acid binding, and heterocyclic compound binding, while the functions of the E. faecalis DEGs in the 48 h triple-species biofilm were mainly annotated to establishment of localization, organic substance transport, transmembrane transport, catalytic complex, transporter activity, and transmembrane transport activity (Fig. 5D and E; Table S3). According to the Kyoto Encyclopedia of Genes and Genomes (KEGG) enrichment analysis, more DEGs of E. faecalis primarily involved in membrane transport, glycan biosynthesis and metabolism, and carbohydrate metabolism were upregulated in 24 h triple-species biofilm, consistent with the accumulation of biomass in the triple-species biofilm in this initial phase (Fig. 5F; Table S4). In comparison, the number of DEGs of E. faecalis involved in membrane transport, glycan biosynthesis and metabolism, and carbohydrate metabolism was reduced in the 48 h triple-species biofilm, which may be related to nutrient depletion in the mature phase (Fig. 5G and Table S4). In summary, the GO and KEGG analyses strongly suggest that E. faecalis establishes dominance in triple-species biofilms by upregulating the nutrient acquisition and carbohydrate metabolism pathways.

In terms of nutrient transport, the expression of 2 DEGs (*ef0892* and *ef0893*) involved in amino acid transport was upregulated 6.3-fold at 24 h (Table 1). The expression of 7 DEGs (*gltA*, *glnA*, *gdhA*, *glmS*, *purF*, *carB*, and *pyraA*) involved in the metabolism and catalysis in the glutamine biosynthesis pathway increased 2.5 to 6.4-fold at 24 h (Table 1). In addition, the expression of 2 DEGs (*ef2180* and *ef2181*) encoding glycosyltransferase 2 family proteins increased 5.3 and 4.5-fold, respectively (Table 1). Furthermore, the expression of some genes encoding glycosyl hydrolases, metallohydrolases, α/β hydrolases, and HAD family hydrolases was upregulated 1.6 to 27.6-fold at 24 h (Table S5). At 48 h, 9 genes involved in sugar transport by the phosphatase transfer system (PTS) were upregulated, especially the genes involved in the transport of mannose (*ef0815*, *ef0816*, and *ef0817*), galactose (*gatA* and *gatC*), and glucose (*ef2438* and *ef0541*), which were upregulated 2.3 to 44.2-fold (Table 1).

**TABLE 1:** DEGs involved in enhanced dominance of E. faecalis

Triple-species biofilm Vs E. faecalis biofilm			
Gene name	Function	Change fold^*a*^	
		24 h	48 h
*ldh-1*			
*ef0255*	L-lactate dehydrogenase	+1.9	+1.9
Glucose			
*ef2438*	PTS transporter subunit IIA components	^*b*^	+8.9
*ef0541*	PTS transporter subunit EIIC components	+3.2	+5.1
Maltose			
*ef0958*	PTS system EIICB components	−2.6	+1.2
*ef0960*	6′-phosphate phosphatase	−2.3	+1.7
Galactose			
*gatA*	PTS system EIIA components	+1.3	+2.3
*gatC*	PTS system EIIC components	−3.8	+3.2
Mannose			
*ef0815*	PTS system EIIAB components	−18.2	+44.2
*ef0816*	PTS system EIIC components	−7.1	+25.1
*ef0817*	PTS system EIID components	−5.0	+2.8
Arginine/Lysine/Histidine			
*ef0892*	Amino acid ABC transporter ATP-binding protein	+6.3	+5.3
*ef0893*	ABC transporter permease subunit	+6.3	+1.7
Glutamine metabolism			
*gltA*	NADPH-dependent glutamate synthase	+3.9	−4.5
*glnA*	Type I glutamate-ammonia ligase	+5.7	+3.0
*gdhA*	NADP-specific glutamate dehydrogenase	+6.4	+2.8
Glutamine catalyzing			
*glmS*	Glutamine-fructose-6-phosphate transaminase	+3.1	+8.3
*purF*	Amidophosphoribosyltransferase	+5.2	+1.4
*carB*	Carbamoyl-phosphate synthase large subunit	+3.6	−1.1
*pyraA*	Carbamoyl phosphate synthase small subunit	+2.5	−1.4
Glycosyltransferase			
*ef2180*	Glycosyltransferase family 2 protein	+5.3	+1.3
*ef2181*	Glycosyltransferase family 2 protein	+4.5	+3.3

a +, up-regulation; −, downregulation.

b , no differential change.

**FIG 6:**
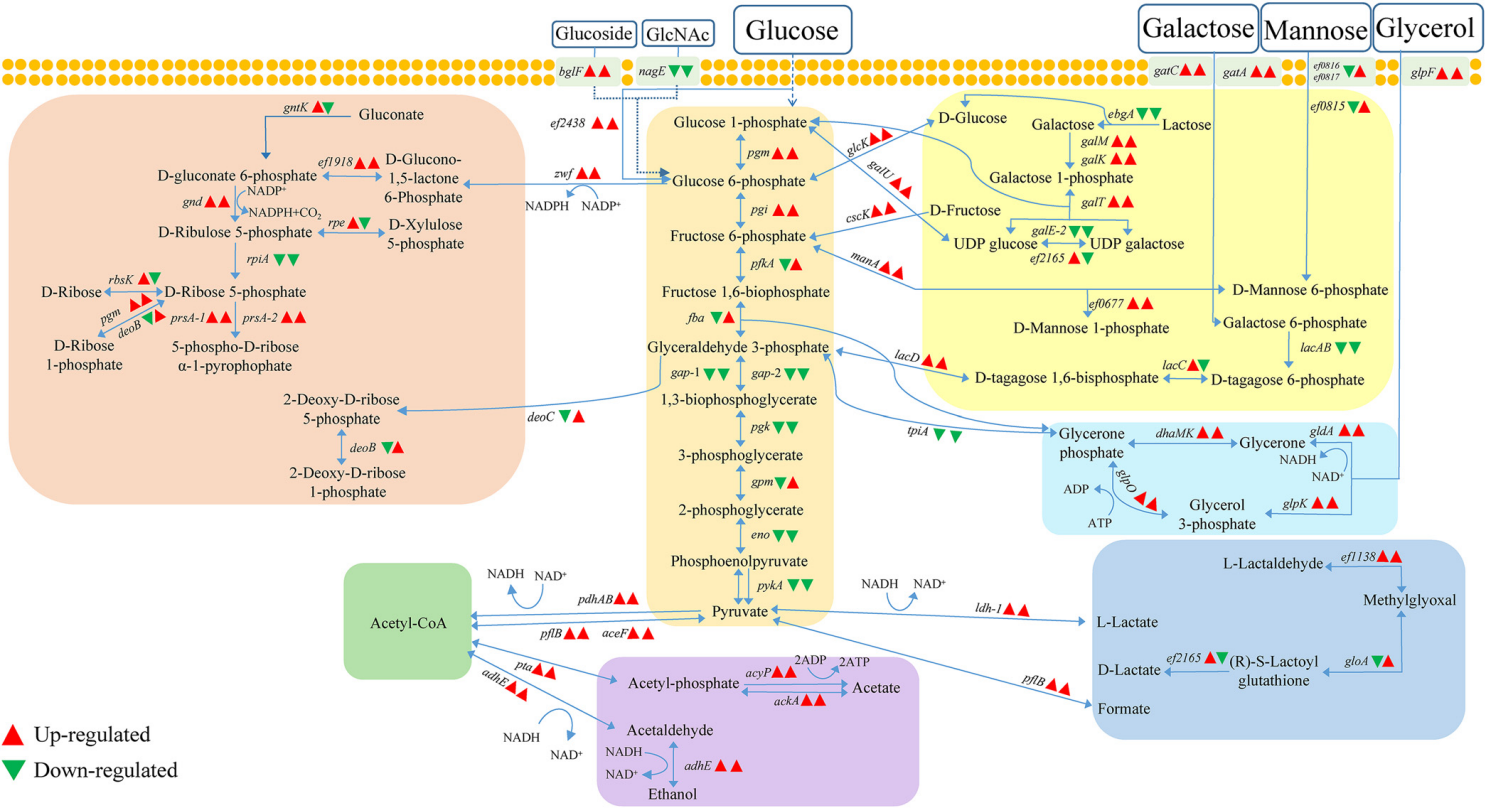
Timely regulation of E. faecalis genes related to central carbon metabolic pathways in the triple-species biofilms. The expression of genes involved in glycolysis/gluconeogenesis, pentose phosphate pathway, lactose transport, acetate, lactate, glycerol, and ethanol metabolism were shown in the diagram. The triangle on the left represents the gene expression pattern at 24 h; the triangle on the right represents the gene expression pattern at 48 h.

In addition, in terms of central carbon metabolism, 47 and 75 genes involved in carbohydrate metabolic process pathways were detected at 24 h and 48 h, respectively (Fig. 6 and Fig. S5; Table S3 and S6). Of these, the *zwf*, *ef1918*, and *gnd genes* involved in the oxidative branch phase of the pentose phosphate pathway (PPP) were upregulated 1.5 to 10.5-fold and 1.3 to 3.8-fold at 24 h and 48 h, respectively. Six genes (*glpF*, *gldA*, *glpK*, *dhaM*, *dhak*, and *glpO*) involved in glycerol metabolism were upregulated 2.7 to 4.0-fold at 24 h and 1.3 to 2.8-fold at 48 h (Table S6).

In addition, our transcriptomic analyses showed that the majority of virulence genes, such as the genes *gelE* encoding gelatinase and *entV* encoding enterocin, were not differentially expressed or were downregulated in the triple-species biofilm (Table S7). Interestingly, the expression of the E. faecalis gene *ldh-1* encoding l-lactate dehydrogenase (LDH) was upregulated 1.9-fold at both 24 h and 48 h in the triple-species biofilm (Table 1). LDH generates lactic acid during the fermentation process. It has been reported that E. faecalis reduces the pH of the polymicrobial environment by generating lactic acid as a “biological weapon” to inhibit the growth of other species and facilitate its own growth (30–33). To test the effect of E. faecalis *ldh-1* upregulation on the pH of the microenvironment in the triple-species biofilm, we determined the pH by pHrodo staining. The results showed that the pH of the microenvironment in the triple-species biofilm decreased with increasing coculture time (Fig. S6).

## DISCUSSION

Multispecies biofilms are generally more tolerant to antimicrobial treatments than the corresponding planktonic cells. Understanding the nature of multispecies biofilms will contribute to the development of novel strategies to treat multispecies biofilm infections. In this pilot study, we used a static biofilm model to investigate the effect of the coexistence of E. faecalis, E. coli O157:H7, and S. enteritidis on triple-species biofilm formation. Given that static biofilms induce a nutrient stress response, they resemble the microenvironment observed during coinfection in *vivo* and in biofilm studies in other environments. We found that the coexistence of E. faecalis with E. coli O157:H7 and S. enteritidis dramatically enhanced the triple-species biofilm biomass, altered its ECM composition, and restructured the biofilm with a tower-like architecture. The triple-species biofilm was dominated by E. faecalis and showed elevated tolerance to disinfectants and antibiotics compared to the E. faecalis mono-species biofilm. Our transcriptome analyses indicated that E. faecalis reprograms its metabolic and regulatory pathways in response to coexistence with E. coli and S. enteritidis.

Generally, bacteria in polybacterial biofilm communities interact with each other synergistically or competitively, resulting in enhanced or decreased biofilm formation, respectively (34, 35). Our results demonstrated that the biofilm biomass was significantly enhanced by different bacterial mixing ratios in the triple-species biofilm model. The biofilm biomass was also significantly increased in the double-species biofilm formed by E. faecalis with E. coli O157:H7 or S. enteritidis, while coculture of E. coli O157:H7 and S. enteritidis dramatically decreased the biofilm biomass. In addition, the triple-species biofilm formed a more complex tower-like architecture than the E. faecalis mono-species biofilm. These findings strongly indicate that intricate interspecies interactions occur when E. faecalis, E. coli O157:H7, and S. enteritidis coexist with each other. Further interactome studies will guide the future investigation of the roles of individual bacterial species in mediating the interspecies interactions.

The principle of competitive exclusion asserts that the adaptive capacity of species to a niche defines its reproduction rates and consequently leads to the dominance or exclusion of the species in multispecies biofilms (8). E. faecalis has a strong adaptive capacity, characterized by persistence in different environments and typical metabolic flexibility (36). We found that E. faecalis dominated the triple-species biofilm, accounting for 93.18% of the bacterial population in the 24 h triple-species biofilm and up to 99.93% in the 48 h triple-species biofilm. This dominance of E. faecalis has also been previously observed in other E. faecalis-harboring multispecies biofilms (37). More importantly, the coexistence of a small portion of E. coli O157:H7 and S. enteritidis with E. faecalis leads to restructuring of the triple-species biofilm into a tower-like form with elevated tolerance to disinfectant and antibiotic treatments. These findings highlight the challenges in the treatment of clinical biofilm infections and the removal of biofilm contamination in food processing.

The formation of a tower-like architecture may promote the stability of triple-species biofilms. Similar tower-like structures biofilms have been observed in E. faecalis biofilms under antibiotic stress and in S. aureus biofilms (38). Additionally, in tower-like biofilms, localization in the top biofilm layers is believed to provide direct growth advantages via continuous supply of and access to resources, especially glucose (11, 39). Nutrient availability is critical for biofilm structure (40). Therefore, access to more nutrients and space for growth may be the top requirement for the survival of each bacterium in resource-limited triple-species biofilms.

The ECM plays a scaffolding role in biofilms (41). The ECM components changed in different phases of triple-species biofilm formation. At 24 h, proteins and polysaccharides dominated the ECM in the triple-species biofilm, while at 48 h, the levels of proteins and polysaccharides decreased, and the eDNA level increased significantly. Similar enhancements in biofilm formation through augmentation of bacterial numbers and eDNA levels were also observed in mixed-species biofilms formed by Staphylococcus epidermidis and Candida albicans (42), as well as Streptococcus mutans and C. albicans (43). Additionally, ECM components can also function as a nutrient pool (44). Under nutritional stress, E. faecalis may utilize ECM components, especially polysaccharides and proteins, as major nutrient sources to acquire fitness benefits (44), consequently leading to a decrease in polysaccharide and protein levels in the ECM of the 48 h triple-species biofilm compared to that of the 24 h biofilm (Fig. 4B). The transcriptomic analyses, showing the upregulation of genes for sugar and amino acid transport in E. faecalis, also indicated the enhanced utilization of polysaccharides and proteins (Table 1). In contrast, the eDNA level in the 48 h triple-species biofilm increased in comparison with that in the 24 h triple-species biofilm (Fig. 4B), consistent with the CLSM results (Fig. 1C). These observations support the previous findings indicating that eDNA functions as a structural component within biofilms, providing stability to the entire structure (45). Furthermore, changes in the ECM can alter the interactions between cells in biofilms, especially between cells of different species (46). These social interactions can lead to changes in the composition and structure of microcolonies in biofilms, thus shaping their overall function, as well as their virulence in the presence of pathogens (9). Thus, such changes in ECM components are important for the clinical treatment of biofilm-related infections.

Competitors in biofilms can utilize the lysate of neighboring cells and the ECM for growth and survival by producing exoenzymes (9, 10). In the triple-species biofilm, E. faecalis used mannose, galactose, and glucose in the ECM as the major carbon sources to engage in central carbon metabolism by upregulating a large number of genes encoding hydrolases, particularly polysaccharide lyases, and genes related to the PTS and ABC transport systems involved in the transport of extracellular sugars and amino acids (47). In addition, upregulation of glycosyltransferase genes may lead to increased catalysis of nucleotide sugars acting as activating monosaccharide donors in E. faecalis, resulting in the production of oligosaccharides/polysaccharides and their derivatives (48). This is similar to the increase in α glucan production caused by the glycosyltransferase GtfB secreted by S. mutans in the enhanced mixed-species biofilms formed by S. mutans and C. albicans (49). Moreover, RNA-seq of E. faecalis in dual-species biofilms (E. faecalis+ E. coli O157:H7) showed increased the expression of genes related to the PTS system and ABC transport system at 24 h (Table S8), indicating that E. faecalis may acquire increased access to more nutrients in dual/multispecies biofilms by regulating the expression of genes related to transport systems. Furthermore, E. faecalis may convert ammonia generated from basic amino acids, including arginine, lysine, and histidine, to active glutamine during the formation of the triple-species biofilm by upregulating the expression of the genes *gltA*, *glnA*, and *gdhA*, and the glutamine is then further catalyzed and processed for aminosugar, purine, and pyrimidine metabolism by upregulating the expression of the genes *glmS*, *purF*, *carB*, and *pyraA* (11). These observations strongly suggest that E. faecalis shows metabolic flexibility and enhanced environmental tolerance.

Competitive interactions between microorganisms in multispecies biofilms are closely linked with their metabolism (50). Nutrient limitations, such as the low glucose levels encountered by species in a multispecies biofilm microenvironment, can lead to changes in the expression of enzyme genes in central carbon metabolic pathways (51). Thus, the capacity of central carbon metabolism under stress conditions defines the fitness and dominance of species in a polymicrobial community. During the formation of the triple-species biofilm (24 h) and in response to nutrient limitation, E. faecalis exhibited rapid upregulation of the expression of enzyme genes (*pgm* and *pgi*) in glycolytic phase I as well as enzyme genes (*zwf*, *ef1918*, and *gnd*) in the oxidative branch phase of the PPP, thereby shifting the carbon flux of sugar metabolism to the PPP, which may reflect the need for E. faecalis in the triple-species biofilm to generate more reducing power for biosynthesis (29). This is similar to the high expression of genes in the PPP of C. albicans in mixed biofilms formed with Pseudomonas aeruginosa (52). Furthermore, E. faecalis can obtain additional carbon sources by using enhanced glycerol metabolic pathways (deoxygenation pathway GldA and phosphorylation pathway GlpK) (33).

Additionally, E. faecalis may establish its dominance by manipulating the microenvironment via the secretion of “biological weapons.” During triple-species biofilm formation, E. faecalis exhibited upregulated expression of *ldh-1*, encoding LDH, which generates lactic acid during the fermentation process. It is known that E. faecalis acidifies the polymicrobial environment by generating lactic acid as a “biological weapon” to inhibit the growth of other species and facilitate its own growth (30, 31, 53). This interference competition with other microorganisms mediated by factors such as secreted metabolites has also been reported in previous studies (4, 54–56). Notably, while acquiring growth advantages, E. faecalis exhibited downregulated expression of virulence genes, including *gelE* encoding a gelatinase with hydrolase function, *entV* encoding an enterocin with bacteriocin function (57), and other virulence genes involved in biofilm formation (Table S7) (1, 58, 59). Acidifying the environment through metabolites and economizing unnecessary and expensive costs, such as the expression of virulence genes, may be beneficial for the dominance of E. faecalis during triple-species biofilm formation.

E. faecalis continuously activates versatile stress response regulators during biofilm formation, enabling its rapid adaptation to its environment (60). During triple-species biofilm formation, genes encoding transcriptional regulators involved in the response, such as GntR, MerR, PadR, ArgR, PerR, AbrB, and LacI, were consistently upregulated in E. faecalis. The key functions of these regulators include the regulation of general metabolism, resistance and detoxification, carbon and nitrogen metabolism, carbon source utilization, arginine metabolism, and oxidative stress (61). These transcriptional responses to a multibacterial environment to enhance the stress response and adaptation capabilities have been described previously (62).

Finally, it has been reported that quorum sensing (QS) can regulate mono- and multispecies biofilm formation by diverse behaviors, such as by controlling the production of matrix components and increasing cell cooperation (63, 64). In E. faecalis, QS systems control major virulence determinants that cause nosocomial infections, including several virulence factors, such as the *cytolysin* operon and the Fsr system (65). The Fsr system indirectly regulates genes that play roles in surface adhesion, autolysis, and biofilm development, including *fsrA*, *fsrB*, *fsrC*, *fsrD*, *gelE*, *sprE*, and *ef1097* (65). However, in our RNA-seq results, we did not observe a significant correlation between the changes in the expression of the Fsr system and the enhancement in biofilm biomass. Although it is possible that QS molecules regulate E. faecalis biofilm formation in an as-yet-unknown indirect manner, this is beyond the focus of our current study. Taken together, the results of our transcriptome analyses indicated that E. faecalis established its dominance in the triple-species biofilm in four possible ways: (i) by enhancing nutrient transport and biosynthesis of amino acids; (ii) by enhancing central carbon metabolism; (iii) by manipulating the microenvironment through “biological weapons”; and (iv) by activating versatile stress response regulators.

**FIG 7:**
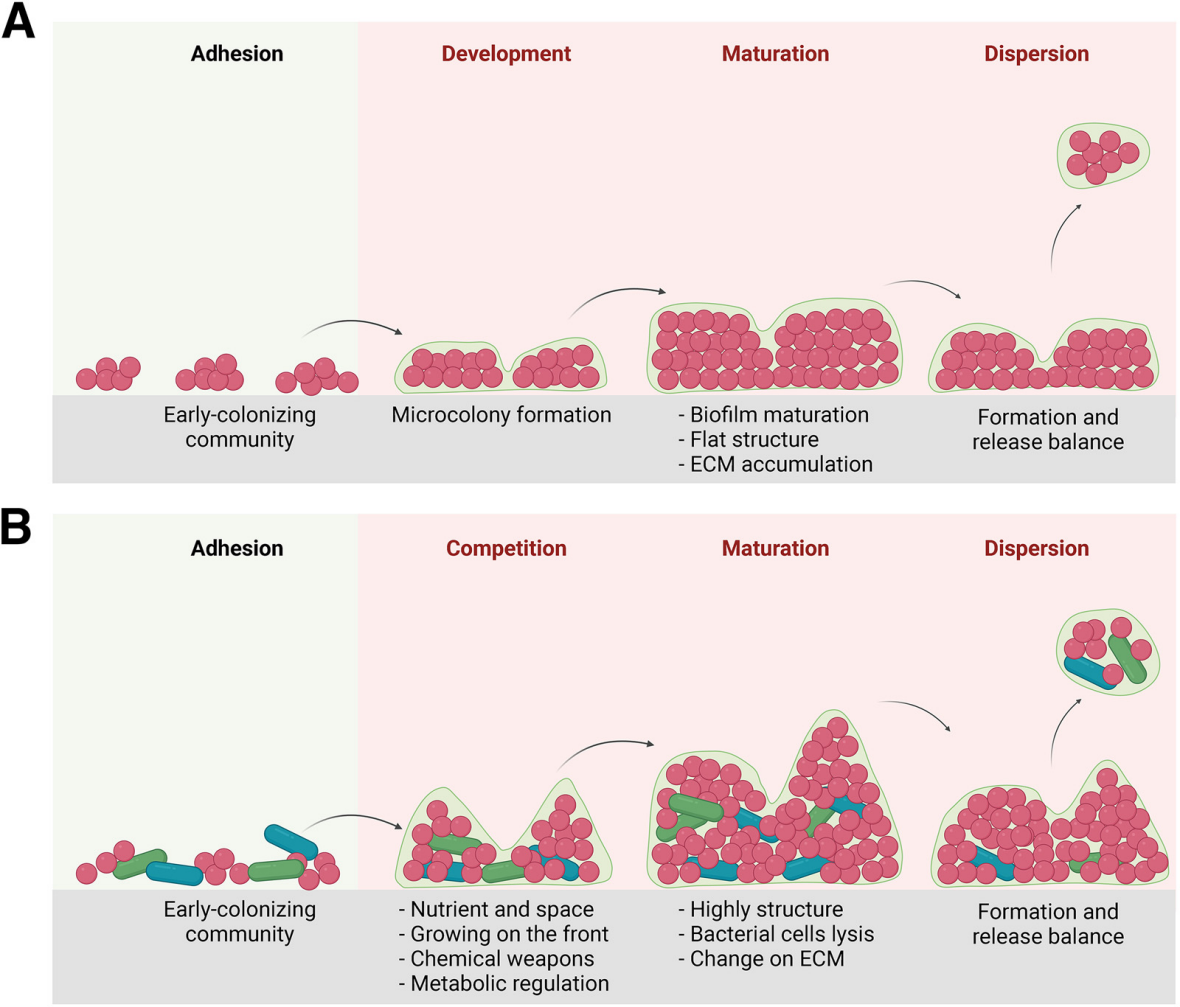
Schematic diagram of the single E. faecalis biofilm and the triple-species biofilm formation. (A) The major processes of E. faecalis biofilm formation, including adhesion, development, maturation, and dispersion phases. (B) The major processes of triple-species biofilm formation, including adhesion, competition, maturation, and dispersion phases. Red represents E. faecalis; blue and green represent E. coli O157:H7 and S. enteritidis, respectively.

In summary, the coexistence of E. faecalis with E. coli and S. enteritidis enhanced triple-species biofilm formation and led to restructuring of the biofilm into a tower-like architecture (Fig. 7). The enhanced biomass production in the triple-species biofilm was an intrinsic community property, with E. faecalis playing a key role in stabilizing interspecies relationships. This pilot study revealing this characteristic of E. faecalis in triple-species biofilms has the potential to reveal amenable targets for the design of targeted antibiofilm inhibitors and to provide novel insights into the treatment of associated biofilm infections and the removal of environmental contamination in food processing. Further study, including testing other bacterial strains with better mono-species biofilm formation capability, performing a time course assay to monitor multispecies biofilm formation, and dissecting the interspecies interaction in triple-species biofilms at the molecular level, will be performed in the future.

## MATERIALS AND METHODS

### Strains and growth conditions.

E. faecalis, E. coli O157:H7, *and* S. enteritidis were inoculated in brain heart infusion (BHI) broth overnight at 37°C. E. faecalis and S. enteritidis strains were isolated from an intensive swine farm by our laboratory. E. coli O157:H7, which is a common foodborne pathogen, was purchased from the National Center for Medical Culture Collections (CMCC44939).

### Biofilm biomass assay.

The cell density of the original “microbial stock solution” for each of the three bacteria was standardized to 1× 10^8^ CFU/mL in BHI medium containing 2% glucose. Then, for mono-species biofilms, 100 μL of the “microbial stock solution” for each of the three species was separately added into the wells of 96-well microplates. In this pilot study, for dual-species biofilms, 100 μL of “microbial stock solution” for each of the two strains was mixed into the 96-well microplates, resulting in a total volume of 200 μL of solution per well. For the triple-species biofilms with different mixing ratios (1:1:1, 2:1:1, 5:1:1, 10:1:1, and 20:1:1), 100 μL of E. faecalis solution was added into the wells of the 96-well microplates, and then the corresponding volumes of E. coli and S. enteritidis solutions were added into the wells containing 100 μL of E. faecalis solution, resulting in a series of mixed solutions corresponding to the above-mentioned proportions (300, 200, 140, 120, or 110 μL). Biofilms for all the tests in this study were prepared by culturing the corresponding single or mixed microbial solutions for 24 h or 48 h. The wells were gently washed 3 times with phosphate-buffered saline (PBS, pH 7.2). Prior to biofilm staining, the plates were dried at room temperature in an inverted position overnight. Biofilm quantification assays were performed as described previously by Christensen (66). The wells were stained with 200 μL of 0.1% crystal violet solution for 15 min at room temperature. After washing and airdrying the plates, the biofilm-associated dye was solubilized in 200 μL of destaining solution consisting of ethanol and acetone at a ratio of 4:1 (vol/vol) and quantified by measuring the absorbance at 570 nm (OD_570_). An equal volume of BHI containing only 2% glucose was used as the negative control to determine the background absorbance. For the antimicrobial tolerance assay and biofilm clearing assay (67), individual bacteria and bacterial mixtures (1:1:1, vol/vol/vol) were grown in 96-well plates for 24 h and 48 h and then washed 3 times with PBS. Biofilms were treated with a range of concentrations of ampicillin, DNase I, proteinase K, and sodium periodate in a volume of 200 μL for 24 h and sodium hypochlorite in a volume of 200 μL for 5 min. Then, the biofilms were washed, dried, stained, and quantified by measuring the OD_570_. Biofilm biomass assays were performed in four biological replicates and three technical replicates for a total of 12 readings.

### CLSM.

One milliliter of each microbial stock solution was added and mixed into a glass-bottom cell culture dish (NEST, China), resulting in 3 mL of culture for triple-species biofilm formation. One milliliter of E. faecalis stock solution was cultured to form the mono-species biofilm. After incubation for either 24 h or 48 h, the wells were gently washed 3 times with PBS and stained as described below. Live/dead staining of the biofilms was performed with the LIVE/DEAD BacLight Bacterial Viability kit (Invitrogen, USA). The determination of biofilm compositions was carried out according to the study of Tee et al. (67) with minor modifications. First, 1 mL of 1×FilmTracer Sypro Ruby biofilm matrix stain (Thermo Fisher Scientific, USA) was added, and the plates were incubated for 20 min. Sypro Ruby was removed, and the wells were washed twice with filtered PBS. Next, 1 mL of 500 μM propidium iodide (Thermo Fisher Scientific, USA) was added, and the plates were incubated for 5 min. The wells were then washed 3 times with filtered PBS. Finally, 1 mL of 0.1% calcofluor white (Sigma–Aldrich) was added for 30 min. After the final incubation, the wells were washed 3 times with distilled water, and fixation was performed with Vectashield Antifade Mounting Medium (Vector Laboratories, USA). Microscopic observation and image acquisition were performed using a Zeiss LSM800 confocal microscope (Zeiss, Germany). Images were analyzed with Zen Black software (Zeiss, Germany). At least three independent experiments were performed on different days, and the images displayed are representative images.

### Visualization of biofilm pH with fluorescence microscopy.

The biofilm used for visualization of biofilm pH was prepared following the same protocol as that used for CLSM and then stained using the method described in the study by Katherine et al. (68) with minor modifications. After incubating for 12, 24, 36, and 48 h, 1 μM pHrodo Red Dextran (Molecular Probes, Invitrogen, USA) was added to the wells, and the mixture was incubated at room temperature in the dark for 3 h. The biofilms were then washed with PBS three times and stained with SYTO9 (Molecular Probes, Invitrogen, USA) diluted in PBS at room temperature in the dark for 15 min. After the final incubation, the wells were washed 3 times with distilled water and then affixed with Vectashield Antifade Mounting Medium (Vector Laboratories, USA). Fluorescence microscopy was performed using 10× objective magnification on an Invitrogen EVOS M5000 fluorescence microscopic imaging system. At least three independent experiments were performed on different days, and the images displayed are representative images.

### SEM.

Then, 0.5 mL of each microbial stock solution was added and mixed into 24-well plates with Aclar coupons (Electron Microscopy Sciences, USA), resulting in 1.5 mL of cultures for triple-species biofilm formation. E. faecalis stock solution (0.5 mL) was cultured under the same conditions to form the mono-species biofilm. The biofilm cultured for either 24 h or 48 h was used for SEM preparation and observation following the standard protocol for SEM. Briefly, the coupons were washed 3 times with PBS and then subjected to fixation in 2.5% glutaraldehyde overnight. Next, fixed samples were washed 3 times with distilled water and then dehydrated using a graded ethanol series (25%, 50%, 75%, 85%, 95% [2×] and 100% [2×]), processed for 15 min each time. After drying at room temperature, the samples were sputter-coated with a layer of gold/platinum (CRESSINGTON, the UK). Specimens were evaluated with an FEI Q45 SEM (Thermo Fisher Scientific, USA). At least three independent experiments were performed on different days, and the images displayed are representative images.

### CFU enumeration.

Biofilms were prepared in the same manner as the sample for the biofilm biomass assay and washed gently 3 times with PBS to remove any unadhered bacterial cells. The bacterial cell aggregates were collected in PBS, vortex shaken for 5 min, and sonicated for 10 min to thoroughly break them up. Then, bacterial cells were diluted and plated onto BHI medium and the corresponding selective media, including Pfizer (E. faecalis), Hektoen Enteric (S. enteritidis), and CT-SMAC (E. coli O157:H7) media, after which CFU were enumerated. The CFU enumeration assay was performed in four biological replicates and three technical replicates for a total of 12 readings.

### Extraction and quantification of ECM components.

Biofilm ECM crude samples were extracted as previously described by Ramirez et al. (69) with minor modifications. One and a half milliliters of each microbial stock solution was mixed together into a 6-well plate, resulting in a 4.5-mL culture for triple-species biofilm formation. One milliliter of E. faecalis stock solution was cultured to form a mono-species biofilm for either 24 h or 48 h. Biofilms were washed 3 times with PBS, collected and suspended in 10 mL of PBS, and vortexed for 5 min. Then, the samples were sonicated in an ultrasonic ice-water bath for 45 min, including three rounds of 15 min of ultrasonication (5 sec ultrasonic pulses with a 20% amplitude and 5 sec intervals) with interval of 5 min, followed by vortexing for 5 min and centrifugation at 3500 rpm for 15 min (Heraeus Multifuge X1R, Thermo Fisher Scientific, USA). The supernatant fraction was recovered and filtered through 0.2 μm acrodisc syringe filters with Supor Membrane (Pall Life Sciences, USA). Next, total exopolysaccharides and eDNA were obtained and quantified in accordance with the method described by T Ramirez et al. The Bradford Protein assay kit (Solarbio, China) was used to quantify the concentration of proteins in the supernatant filtrate. ECM component quantification was performed in four biological replicates and three technical replicates, resulting in a total of 12 readings.

### Transcriptome analysis using RNA-seq.

RNA-seq was performed by Personal Biotechnology Company (Shanghai, China) using the Pacific Biosciences platform and the Illumina NovaSeq platform. Three biological replicate biofilms were prepared in the same way as sample preparation for ECM component extraction and quantification. RNA from E. faecalis in the mono-species biofilm and triple-species biofilm samples was extracted using TRIzol Reagent (Invitrogen Life Technologies, USA). RNA purity was determined using a NanoDrop spectrophotometer (Thermo Fisher Scientific, USA), and RNA integrity was assessed using the RNA Nano 6000 assay kit for the Bioanalyzer 2100 system (Agilent Technologies, USA). A portion of the RNA was used for qPCR. Library preparation was completed using the NEBNext Ultra II RNA Library Prep kit for Illumina (NEB, USA) following the manufacturer’s recommendations. The library was then sequenced on the NovaSeq 6000 platform (Illumina). Cutadapt software v1.15 was used to filter the sequencing data to obtain high-quality sequences for further analysis. The filtered reads were mapped to the reference genome (E. faecalis V583) using HISAT2 v2.0.5, followed by quantification of the gene expression level. HTSeq statistics were used to compare the read count values for each gene as the original expression level of the gene. Then, FPKM was used to standardize the expression levels. Differential expression analysis of three biological replicates was performed using DESeq (1.30.0) with screened conditions as follows: expression difference multiple |log2FoldChange| ≥1 and corrected *P* value < 0.05. GO enrichment analysis of DEGs was performed by topGO. ClusterProfiler (3.4.4) software was used to test the statistical enrichment of DEGs in the KEGG pathways.

### Verification of DEGs by qPCR.

A subset of genes that were differentially regulated in the triple-species biofilm (i.e., *ebpA*, *ebpB*, *gnd*, *gap-1*, and *gpm*) was verified by qRT–PCR using ChamQ Universal SYBR qPCR Master Mix (number Q711-02, Vazyme Biotech Co., Ltd., China). Partial primers were designed using Primer v5.0 software (Table 2), and melt curve analysis was performed at the end of each amplification run to verify signal specificity. The results are presented as the relative expression levels normalized to the level of the housekeeping gene 16S rRNA. The assay was performed in three biological replicates and three technical replicates.

**TABLE 2:** qPCR primers used in this study

Primers	Sequence (5′–3′)	Length(bp)
*ebpA*		
F	CAACAACACCAGGGCTTTTTG	101
R	ACCGGACCAGTCAACGACTAAG	
*ebpB*		
F	CGTACAGGCGGCAAGTCTTT	101
R	AGGTATTCCCCCGCTTGATTT	
*gnd* (29)		
F	CCTGCAGCCTTTAACTTTGC	101
R	GCAAAACCTTGCGCATAACT	
*gap-1* (29)		
F	CGTGACAGCCACTGAAAATG	100
R	TCCGTTTTCTTTAACCCAAGG	
*gpm*		
F	TTTTGATGGCACGAGTT	126
R	GATGGGCTGACGTAGAT	

### Statistical analysis.

Significant differences between experimental groups were determined using a paired Student's *t* test in GraphPad Prism v8.0.2 software. For all analyses, a *P* value of <0.05 was considered to indicate statistical significance. “*” indicates 0.01 < *P < *0.05, “**” indicates 0.001 < *P < *0.01, and “***” indicates *P < *0.001.

### Data accessibility.

The accession number for the RNA-seq and related metadata reported in this paper is NCBI: PRJNA755819. Other data that support the findings of this study are available from the corresponding author upon reasonable request.

## References

[1] Guiton P, Hung C, Kline K, Roth R, Kau A, Hayes E, Heuser J, Dodson K, Caparon M, Hultgren S. 2009. Contribution of autolysin and Sortase a during Enterococcus faecalis DNA-dependent biofilm development. Infect Immun 77:3626–3638. doi:10.1128/IAI.00219-09. 19528211 PMC2738007

[2] Lewis K. 2001. Riddle of biofilm resistance. Antimicrob Agents Chemother 45:999–1007. doi:10.1128/AAC.45.4.999-1007.2001. 11257008 PMC90417

[3] Aussel L, Beuzón CR, Cascales E. 2016. Meeting report: adaptation and communication of bacterial pathogens. Virulence 7:481–490. doi:10.1080/21505594.2016.1152441. 26890494 PMC4871651

[4] Gabrilska R, Rumbaugh K. 2015. Biofilm models of polymicrobial infection. Future Microbiol 10:1997–2015. doi:10.2217/fmb.15.109. 26592098 PMC4944397

[5] Elias S, Banin E. 2012. Multi-species biofilms: living with friendly neighbors. FEMS Microbiol Rev 36:990–1004. doi:10.1111/j.1574-6976.2012.00325.x. 22229800

[6] Orazi G, O'Toole G. 2019. “It takes a village”: mechanisms underlying antimicrobial recalcitrance of polymicrobial biofilms. J Bacteriology 202:e00530-19. 10.1128/JB.00530-19 PMC6932244 31548277

[7] Liu W, Russel J, Burmølle M, Sørensen SRJ, Madsen JS. 2018. Micro-scale intermixing: a requisite for stable and synergistic co-establishment in a four-species biofilm. ISME J 12:1940–1951. doi:10.1038/s41396-018-0112-2. 29670216 PMC6052071

[8] Ursell T. 2021. Structured environments foster competitor coexistence by manipulating interspecies interfaces. PLoS Comput Biol 17:e1007762. doi:10.1371/journal.pcbi.1007762. 33412560 PMC7790539

[9] Nadell C, Drescher K, Foster K. 2016. Spatial structure, cooperation and competition in biofilms. Nat Rev Microbiol 14:589–600. doi:10.1038/nrmicro.2016.84. 27452230

[10] Burmølle M, Ren D, Bjarnsholt T, Sørensen S. 2014. Interactions in multispecies biofilms: do they actually matter? Trends Microbiol 22:84–91. doi:10.1016/j.tim.2013.12.004. 24440178

[11] Liu W, Jacquiod S, Brejnrod A, Russel J, Burmølle M, Sørensen S. 2019. Deciphering links between bacterial interactions and spatial organization in multispecies biofilms. ISME J 13:3054–3066. doi:10.1038/s41396-019-0494-9. 31455806 PMC6864094

[12] Willett J, Dale J, Kwiatkowski L, Powers J, Korir M, Kohli R, Barnes A, Dunny G. 2021. Comparative biofilm assays using Enterococcus faecalis OG1RF identify new determinants of biofilm formation. mBio 12:e0101121. doi:10.1128/mBio.01011-21. 34126766 PMC8262879

[13] Dunny GM, Hancock LE, Shankar N. 2014. Enterococcal biofilm structure and role in colonization and disease. *In* Gilmore MS, Clewell DB, Ike Y, Shankar N (ed), Enterococci: from commensals to leading causes of drug resistant infection. Massachusetts Eye and Ear Infirmary, Boston, MA. 24649508

[14] Tien B, Goh H, Chong K, Bhaduri-Tagore S, Holec S, Dress R, Ginhoux F, Ingersoll M, Williams R, Kline K. 2017. Enterococcus faecalis promotes innate immune suppression and polymicrobial catheter-associated urinary tract infection. Infection Immunity 85:e00378-17. 28893918 10.1128/IAI.00378-17 PMC5695114

[15] Laganenka L, Sourjik V. 2018. Autoinducer 2-dependent Escherichia coli biofilm formation is enhanced in a dual-species coculture. Applied Environ Microbiol 84:e02638-17. 10.1128/AEM.02638-17 PMC5812939 29269492

[16] Keogh D, Tay W, Ho Y, Dale J, Chen S, Umashankar S, Williams RBH, Chen SL, Dunny GM, Kline KA. 2016. Enterococcal metabolite cues facilitate interspecies niche modulation and polymicrobial infection. Cell Host Microbe 20:493–503. doi:10.1016/j.chom.2016.09.004. 27736645 PMC5076562

[17] Ch'ng J, Chong K, Lam L, Wong J, Kline K. 2019. Biofilm-associated infection by enterococci. Nat Rev Microbiol 17:82–94. doi:10.1038/s41579-018-0107-z. 30337708 PMC13729018

[18] Vergadi E, Maraki S, Dardamani E, Ladomenou F, Galanakis E. 2021. Polymicrobial gastroenteritis in children. Acta Paediatr 110:2240–2245. doi:10.1111/apa.15854. 33755990

[19] Fourcade C, Canini L, Lavigne J, Sotto A. 2015. A comparison of monomicrobial versus polymicrobial Enterococcus faecalis bacteriuria in a French University Hospital. Eur J Clin Microbiol Infect Dis 34:1667–1673. doi:10.1007/s10096-015-2403-0. 25987245

[20] Liu Y, Cao Y, Wang T, Dong Q, Li J, Niu C. 2019. Detection of 12 common food-borne bacterial pathogens by TaqMan real-time PCR using a single set of reaction conditions. Front Microbiol 10:222. doi:10.3389/fmicb.2019.00222. 30814987 PMC6381072

[21] Friesema IH, Slegers-Fitz-James IA, Wit B, Franz E. 2022. Surveillance and characteristics of food-borne outbreaks in the Netherlands, 2006 to 2019. Euro Surveillance 27:2100071. 35057901 10.2807/1560-7917.ES.2022.27.3.2100071 PMC8804662

[22] Long NS, Wells JE, Berry ED, Legako JF, Woerner DR, Loneragan GH, Broadway PR, Carroll JA, Sanchez N, Fernando SC, Bacon CM, Helmuth CL, Smock TM, Manahan JL, Hoffman AA, Hales KE. 2022. Metaphylactic antimicrobial effects on occurrences of antimicrobial resistance in Salmonella enterica, Escherichia coli and Enterococcus spp. measured longitudinally from feedlot arrival to harvest in high-risk beef cattle. J Appl Microbiol 133:1940–1955. doi:10.1111/jam.15691. 35766106 PMC9546201

[23] Cho S, Jackson CR, Frye JG. 2020. The prevalence and antimicrobial resistance phenotypes of Salmonella, Escherichia coli and Enterococcus sp. in surface water. Lett Appl Microbiol 71:3–25. doi:10.1111/lam.13301. 32304575

[24] Namvar A, Warriner K. 2006. Application of enterobacterial repetitive intergenic consensus-polymerase chain reaction to trace the fate of generic Escherichia coli within a high capacity pork slaughter line. Int J Food Microbiol 108:155–163. doi:10.1016/j.ijfoodmicro.2005.11.006. 16386814

[25] Soares-Santos V, Barreto AS, Semedo-Lemsaddek T. 2015. Characterization of Enterococci from food and food-related settings. J Food Prot 78:1320–1326. doi:10.4315/0362-028X.JFP-14-419. 26197283

[26] Yuan L, Hansen M, Røder H, Wang N, Burmølle M, He G. 2020. Mixed-species biofilms in the food industry: current knowledge and novel control strategies. Crit Rev Food Sci Nutr 60:2277–2293. doi:10.1080/10408398.2019.1632790. 31257907

[27] Giaouris E, Heir E, Desvaux M, Hébraud M, Møretrø T, Langsrud S, Doulgeraki A, Nychas G, Kačániová M, Czaczyk K, Ölmez H, Simões M. 2015. Intra- and inter-species interactions within biofilms of important foodborne bacterial pathogens. Front Microbiol 6:841. doi:10.3389/fmicb.2015.00841. 26347727 PMC4542319

[28] Mohamed J, Huang D. 2007. Biofilm formation by enterococci. J Med Microbiol 56:1581–1588. doi:10.1099/jmm.0.47331-0. 18033823

[29] Muller C, Cacaci M, Sauvageot N, Sanguinetti M, Rattei T, Eder T, Giard J-C, Kalinowski J, Hain T, Hartke A. 2015. the intraperitoneal transcriptome of the opportunistic pathogen Enterococcus faecalis in mice. PLoS One 10:e0126143. doi:10.1371/journal.pone.0126143. 25978463 PMC4433114

[30] Murakami N, Oba M, Iwamoto M, Tashiro Y, Noguchi T, Bonkohara K, Abdel-Rahman M, Zendo T, Shimoda M, Sakai K, Sonomoto K. 2016. L-Lactic acid production from glycerol coupled with acetic acid metabolism by Enterococcus faecalis without carbon loss. J Biosci Bioeng 121:89–95. doi:10.1016/j.jbiosc.2015.05.009. 26168904

[31] Yang Q, Lü Y, Zhang M, Gong Y, Li Z, Tran N, He Y, Zhu C, Lu Y, Zhang Y, Li S. 2019. Lactic acid bacteria, Enterococcus faecalis Y17 and Pediococcus pentosaceus G11, improved growth performance, and immunity of mud crab (Scylla paramamosain). Fish Shellfish Immunol 93:135–143. doi:10.1016/j.fsi.2019.07.050. 31326583

[32] Rana N, Sauvageot N, Laplace J, Bao Y, Nes I, Rincé A, Posteraro B, Sanguinetti M, Hartke A. 2013. Redox balance via lactate dehydrogenase is important for multiple stress resistance and virulence in Enterococcus faecalis. Infect Immun 81:2662–2668. doi:10.1128/IAI.01299-12. 23649090 PMC3719593

[33] Doi Y. 2018. Lactic acid fermentation is the main aerobic metabolic pathway in Enterococcus faecalis metabolizing a high concentration of glycerol. Appl Microbiol Biotechnol 102:10183–10192. doi:10.1007/s00253-018-9351-4. 30232536

[34] Celiker H, Gore J. 2013. Cellular cooperation: insights from microbes. Trends Cell Biol 23:9–15. doi:10.1016/j.tcb.2012.08.010. 22999189

[35] Lasa I, Solano C. 2018. Polymicrobial infections: do bacteria behave differently depending on their neighbours? Virulence 9:895–897. doi:10.1080/21505594.2018.1426520. 29405827 PMC5955476

[36] Gaca A, Lemos J. 2019. Adaptation to adversity: the intermingling of stress tolerance and pathogenesis in Enterococci. Microbiology Molecular Biology Rev 83:e00008-19. 10.1128/MMBR.00008-19 PMC6710459 31315902

[37] Chávez de Paz L, Davies J, Bergenholtz G, Svensäter G. 2015. Strains of Enterococcus faecalis differ in their ability to coexist in biofilms with other root canal bacteria. Int Endod J 48:916–925. doi:10.1111/iej.12501. 26172346

[38] Dale J, Nilson J, Barnes A, Dunny G. 2017. Enterococcus faecalisRestructuring of biofilm architecture in response to antibiotic-induced stress. NPJ Biofilms Microbiomes 3:15. doi:10.1038/s41522-017-0023-4. 28685097 PMC5493694

[39] Chitlapilly Dass S, Wang R. 2022. Biofilm through the looking glass: a microbial food safety perspective. Pathogens 11:346. doi:10.3390/pathogens11030346. 35335670 PMC8954374

[40] Nadell C, Xavier J, Foster K. 2009. The sociobiology of biofilms. FEMS Microbiol Rev 33:206–224. doi:10.1111/j.1574-6976.2008.00150.x. 19067751

[41] Karygianni L, Ren Z, Koo H, Thurnheer T. 2020. Biofilm matrixome: extracellular components in structured microbial communities. Trends Microbiol 28:668–681. doi:10.1016/j.tim.2020.03.016. 32663461

[42] Pammi M, Liang R, Hicks J, Mistretta T, Versalovic J. 2013. Biofilm extracellular DNA enhances mixed species biofilms of Staphylococcus epidermidis and Candida albicans. BMC Microbiol 13:257. doi:10.1186/1471-2180-13-257. 24228850 PMC3833181

[43] Khoury Z, Vila T, Puthran T, Sultan A, Montelongo-Jauregui D, Melo M, Jabra-Rizk M. 2020. The role of Candida albicans secreted polysaccharides in augmenting adherence and mixed biofilm formation: in vitro and in vivo studies. Front Microbiol 11:307. doi:10.3389/fmicb.2020.00307. 32256460 PMC7093027

[44] Dragoš A, Kovács Á. 2017. The peculiar functions of the bacterial extracellular matrix. Trends Microbiol 25:257–266. doi:10.1016/j.tim.2016.12.010. 28089324

[45] Ibáñez de Aldecoa A, Zafra O, González-Pastor J. 2017. Mechanisms and regulation of extracellular DNA release and its biological roles in microbial communities. Front Microbiol 8:1390. doi:10.3389/fmicb.2017.01390. 28798731 PMC5527159

[46] Bowen W, Burne R, Wu H, Koo H. 2018. Oral biofilms: pathogens, matrix, and polymicrobial interactions in microenvironments. Trends Microbiol 26:229–242. doi:10.1016/j.tim.2017.09.008. 29097091 PMC5834367

[47] Fan T, Goeser L, Naziripour A, Redinbo M, Hansen J. 2019. Enterococcus faecalis gluconate phosphotransferase system accelerates experimental colitis and bacterial killing by macrophages. Infect Immun 87:e00080-19. doi:10.1128/IAI.00080-19. 31036600 PMC6589050

[48] Zhao L, Ma Z, Yin J, Shi G, Ding Z. 2021. Biological strategies for oligo/polysaccharide synthesis: biocatalyst and microbial cell factory. Carbohydr Polym 258:117695. doi:10.1016/j.carbpol.2021.117695. 33593568

[49] Sadiq F, Hansen M, Burmølle M, Heyndrickx M, Flint S, Lu W, Chen W, Zhang H. 2022. Towards understanding mechanisms and functional consequences of bacterial interactions with members of various kingdoms in complex biofilms that abound in nature. FEMS Microbiology Rev 46:fuac024. doi:10.1093/femsre/fuac024.

[50] Watkins E, Maiden M, Gupta S. 2016. Metabolic competition as a driver of bacterial population structure. Future Microbiol 11:1339–1357. doi:10.2217/fmb-2016-0079. 27660887

[51] Marmion M, Macori G, Ferone M, Whyte P, Scannell A. 2022. Survive and thrive: control mechanisms that facilitate bacterial adaptation to survive manufacturing-related stress. Int J Food Microbiol 368:109612. doi:10.1016/j.ijfoodmicro.2022.109612. 35278797

[52] Trejo-Hernández A, Andrade-Domínguez A, Hernández M, Encarnación S. 2014. Interspecies competition triggers virulence and mutability in Candida albicans-Pseudomonas aeruginosa mixed biofilms. ISME J 8:1974–1988. doi:10.1038/ismej.2014.53. 24739628 PMC4184018

[53] Segarra R, Booth M, Morales D, Huycke M, Gilmore M. 1991. Molecular characterization of the Enterococcus faecalis cytolysin activator. Infect Immun 59:1239–1246. doi:10.1128/iai.59.4.1239-1246.1991. 1900808 PMC257833

[54] Raffatellu M. 2018. Learning from bacterial competition in the host to develop antimicrobials. Nat Med 24:1097–1103. doi:10.1038/s41591-018-0145-0. 30082869

[55] Kreth J, Merritt J, Shi W, Qi F. 2005. Competition and coexistence between Streptococcus mutans and Streptococcus sanguinis in the dental biofilm. J Bacteriol 187:7193–7203. doi:10.1128/JB.187.21.7193-7203.2005. 16237003 PMC1272965

[56] Ballén V, Ratia C, Cepas V, Soto S. 2020. Enterococcus faecalis inhibits growth in polymicrobial biofilms in a glucose-enriched medium. Biofouling 36:846–861. doi:10.1080/08927014.2020.1824272. 32972252

[57] Wirth R. 1994. The sex pheromone system of Enterococcus faecalis. More than just a plasmid-collection mechanism? Eur J Biochem 222:235–246. doi:10.1111/j.1432-1033.1994.tb18862.x. 8020463

[58] Afonina I, Lim X, Tan R, Kline KA. 2018. Planktonic interference and biofilm alliance between aggregation substance and endocarditis- and biofilm-associated pili in Enterococcus faecalis. J Bacteriology 200:e00361-18. doi:10.1128/JB.00361-18. PMC6256026 30249706

[59] Kristich C, Nguyen V, Le T, Barnes A, Grindle S, Dunny G. 2008. Development and use of an efficient system for random mariner transposon mutagenesis to identify novel genetic determinants of biofilm formation in the core Enterococcus faecalis genome. Appl Environ Microbiol 74:3377–3386. doi:10.1128/AEM.02665-07. 18408066 PMC2423031

[60] Salze M, Giard J, Riboulet-Bisson E, Hain T, Rincé A, Muller C. 2020. Identification of the general stress stimulon related to colonization in Enterococcus faecalis. Arch Microbiol 202:233–246. doi:10.1007/s00203-019-01735-8. 31599337

[61] Ramos J, Martínez-Bueno M, Molina-Henares A, Terán W, Watanabe K, Zhang X, Gallegos M, Brennan R, Tobes R. 2005. The TetR family of transcriptional repressors. Microbiol Mol Biol Rev 69:326–356. doi:10.1128/MMBR.69.2.326-356.2005. 15944459 PMC1197418

[62] Viçosa G, Botta C, Ferrocino I, Bertolino M, Ventura M, Nero LA, Cocolin L. 2018. Staphylococcus aureus undergoes major transcriptional reorganization during growth with Enterococcus faecalis in milk. Food Microbiol 73:17–28. doi:10.1016/j.fm.2018.01.007. 29526203

[63] Abisado RG, Benomar S, Klaus JR, Dandekar AA, Chandler JR. 2018. Bacterial Quorum Sensing and Microbial Community Interactions. mBio 9:e02331-17. doi:10.1128/mBio.01749-18. 29789364 PMC5964356

[64] O'Loughlin CT, Miller LC, Siryaporn A, Drescher K, Semmelhack MF, Bassler BL. 2013. A quorum-sensing inhibitor blocks Pseudomonas aeruginosa virulence and biofilm formation. Proc Natl Acad Sci USA 110:17981–17986. doi:10.1073/pnas.1316981110. 24143808 PMC3816427

[65] Ali L, Goraya M, Arafat Y, Ajmal M, Chen J, Yu D. 2017. Molecular Mechanism of quorum-sensing in Enterococcus faecalis: its role in virulence and therapeutic approaches. Int J Molecular Sciences 18:960. doi:10.3390/ijms18050960. PMC5454873 28467378

[66] Christensen GD, Simpson WA, Younger JJ, Baddour LM, Barrett FF, Melton DM, Beachey EH. 1985. Adherence of coagulase-negative staphylococci to plastic tissue culture plates: a quantitative model for the adherence of staphylococci to medical devices. J Clin Microbiol 22:996–1006. doi:10.1128/jcm.22.6.996-1006.1985. 3905855 PMC271866

[67] Windham I, Servetas S, Whitmire J, Pletzer D, Hancock R, Merrell D. 2018. Helicobacter pylori biofilm formation is differentially affected by common culture conditions, and proteins play a central role in the biofilm matrix. Applied Environ Microbiol 84. doi:10.1128/AEM.00391-18. PMC6029101 29752266

[68] Rainey K, Michalek SM, Wen ZT, Wu H. 2018. Glycosyltransferase-mediated biofilm matrix dynamics and virulence of Streptococcus mutans. Applied Environ Microbiol 85:e02247-18. 10.1128/AEM.02247-18 PMC6384114 30578260

[69] Ramirez T, Shrestha A, Kishen A. 2019. Inflammatory potential of monospecies biofilm matrix components. Int Endod J 52:1020–1027. doi:10.1111/iej.13093. 30719720

