# Supplementary material for: The Coexistence of Bacterial Species Restructures Biofilm Architecture and Increases Tolerance to Antimicrobial Agents
Source: Microbiol Spectr. 2023 Feb 27;11(2):e03581-22. doi: 10.1128/spectrum.03581-22 (PMC10100793; doi:10.1128/spectrum.03581-22)
Supplement: Supplemental file 1 — Figures S1-S6. Download spectrum.03581-22-s0001.pdf, PDF file, 6.0 MB [file spectrum.03581-22-s0001.pdf]

Supplementary Figures

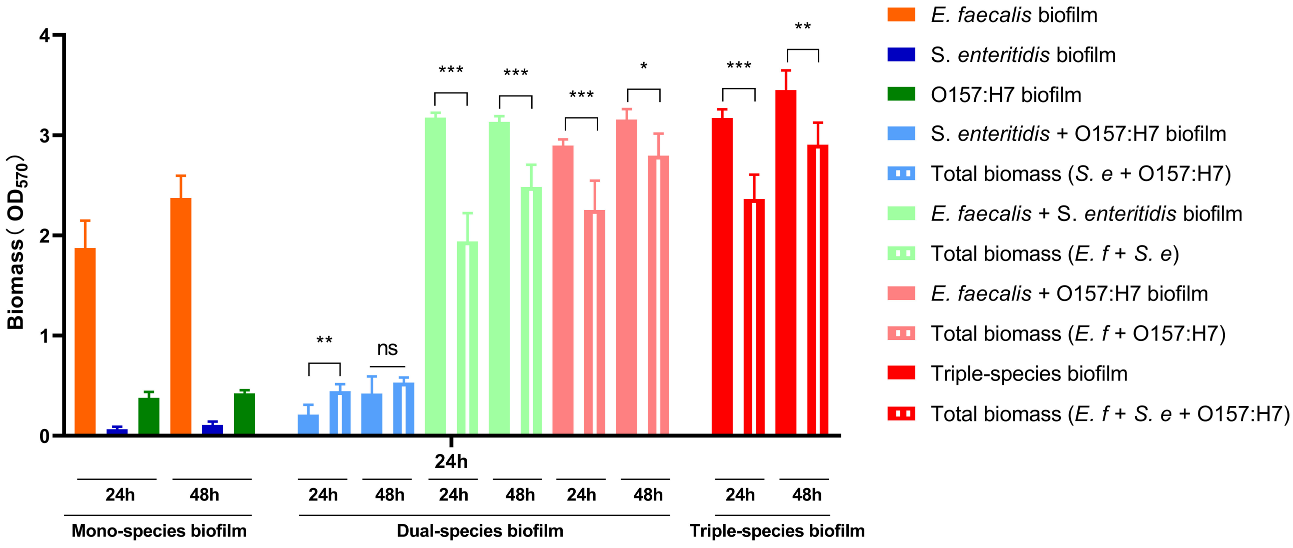

Supplementary Figure S1. Multi-bacterial co-culture enhanced the biofilm formation. Biomass of individual mono-species biofilms, double-species biofilms at a ratio of 1:1, and triple-species biofilms at a ratio of 1:1:1 at 24 and 48 h.

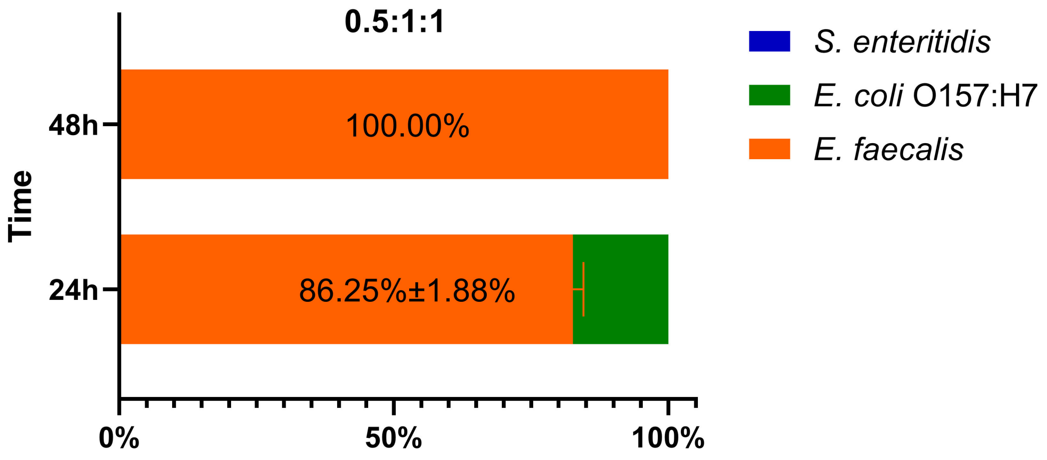

Supplementary Figure S2. Proportion of individual bacteria to the total number of bacteria in the triple-species biofilms at 24 and 48 h. The ratio of initial number for *E. faecalis*, *E. coli* O157:H7, and *S. enteritidis* was 0.5:1:1.

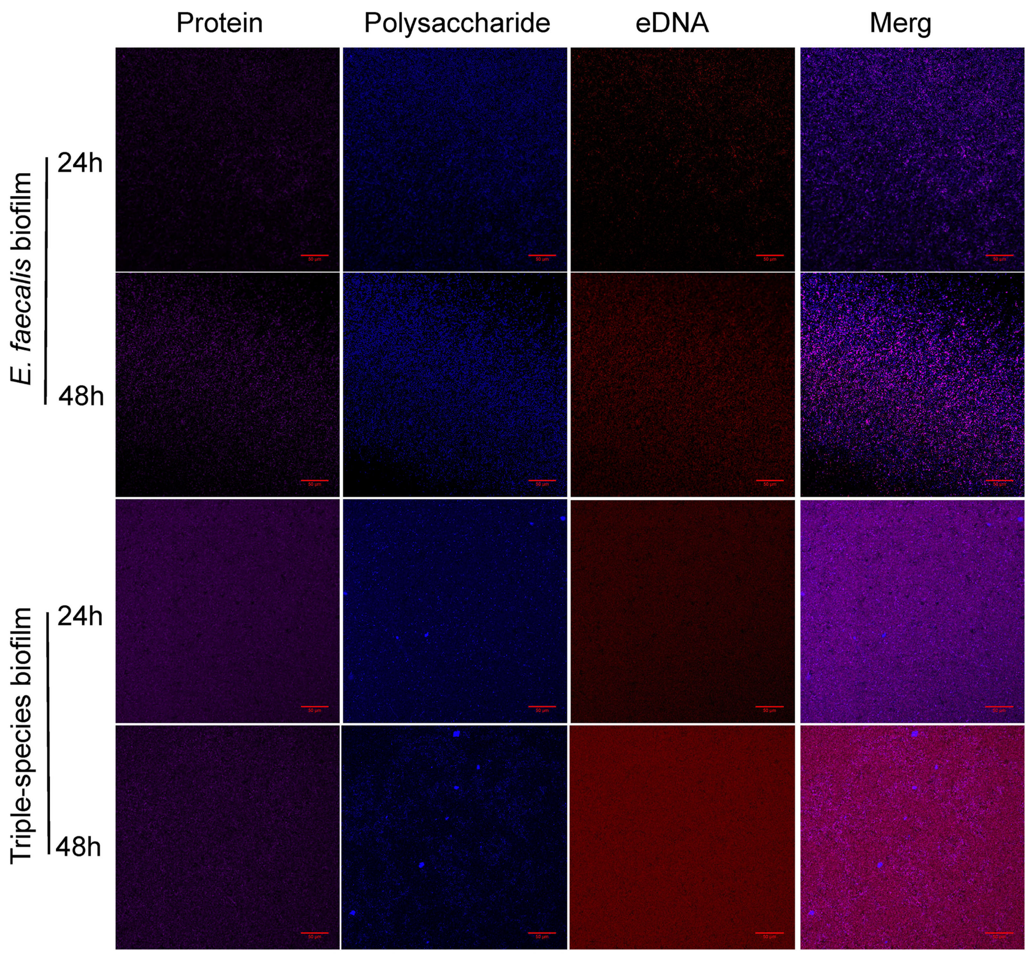

Supplementary Figure S3. Visualization of proteins, polysaccharides, and eDNA in the ECM of biofilms. Representative regions for areas with pronounced biofilm formation are shown. Each dye was captured on a single channel, and the images show a compressed z-stack; merged images at a magnification of ×40 are shown on the far right. The formed biofilms were stained using Sypro Ruby, propidium iodide, and calcofluor white. Sypro Ruby purple for proteins, calcofluor white for polysaccharides, and propidium iodide red for eDNA. The images are representative of at least three independent experiments. Bars, 50 μm.

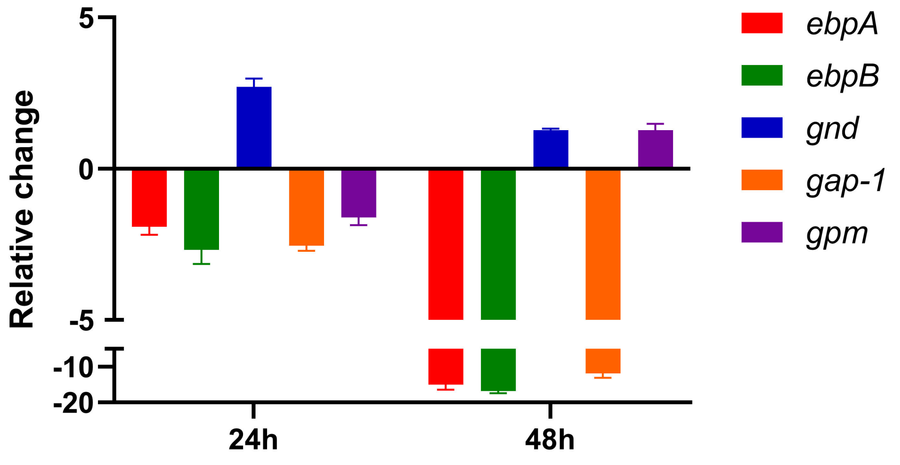

Supplementary Figure S4. qPCR results of partial DEGs. DEGs were verified by qPCR, including *ebpA*, *ebpB*, *gnd*, *gap-1*, and *gpm*. The expression level of mRNA was normalized to the level of 16S rRNA.

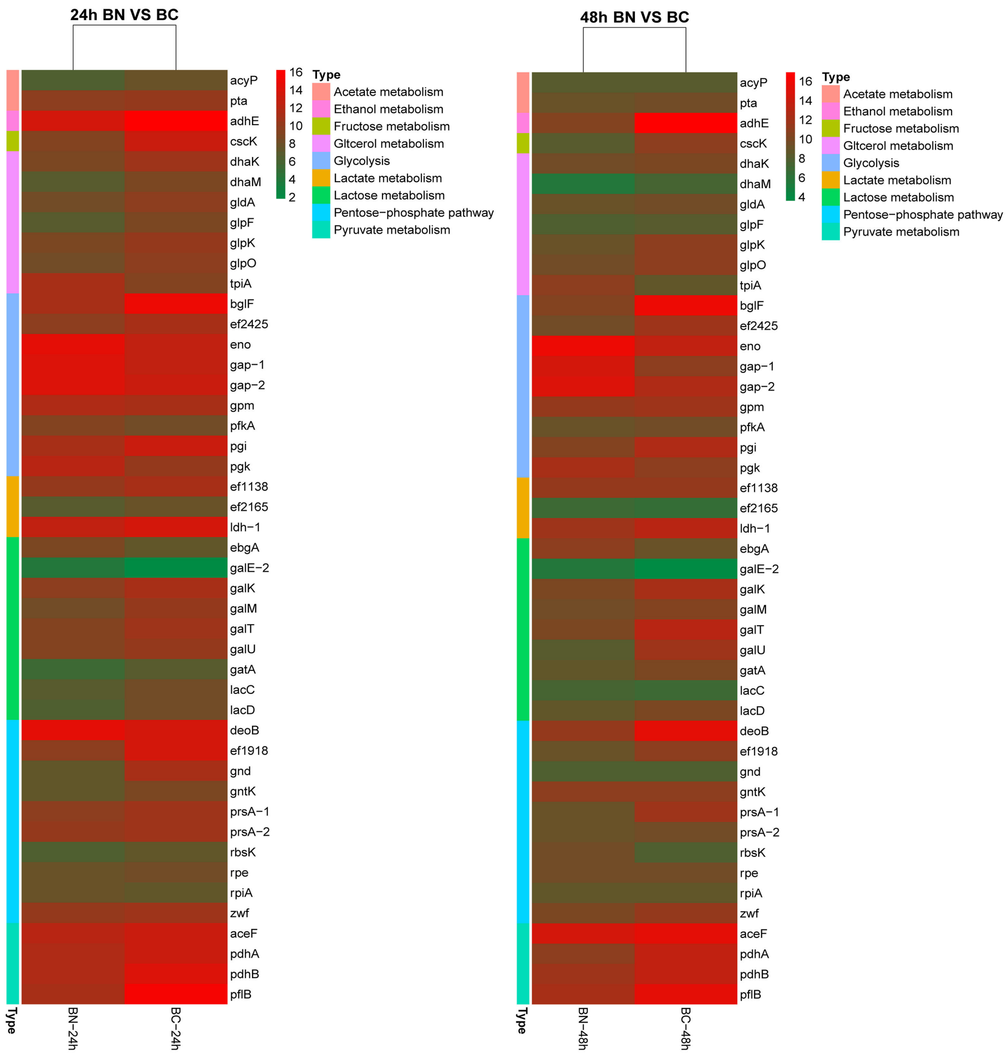

Supplementary Figure S5. Heat map of DEGs related to carbon central metabolism. Central carbon metabolism consists of acetate metabolism, ethanol metabolism, fructose metabolism, glycerol metabolism, glycolysis, lactate metabolism, lactose metabolism, pentose phosphate pathway, and pyruvate metabolism, represented by different color types. BN is *E. faecalis* biofilm, BC is the triple-species biofilm. Red means up-regulation, green means down-regulation.

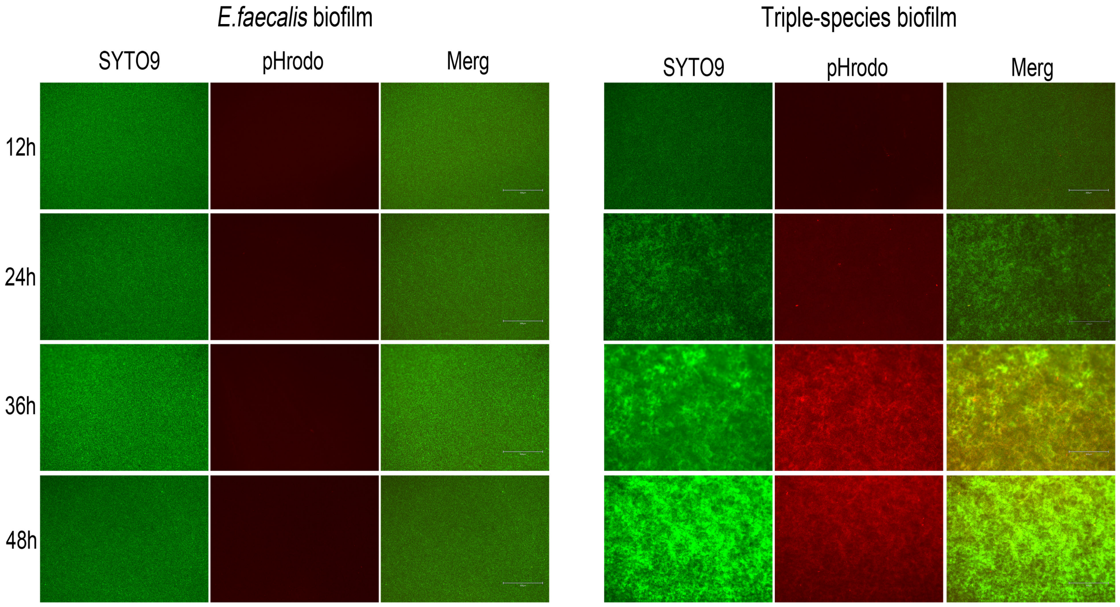

Supplementary Figure S6. Visualization of biofilm pH. After incubation of the biofilm, pHrodo^{TM} Red Dextran and SYTO9 were used to stain successively. SYTO9 green for living cells, and pHrodo red for biofilm pH. Dextran conjugation produces bright red fluorescence in acidic environments. Images are representative of at least three independent experiments. Bars, 300 μm.
